# Polygenic pleiotropy and potential causal relationships between educational attainment, neurobiological profile, and positive psychotic symptoms

**DOI:** 10.1038/s41398-018-0144-4

**Published:** 2018-05-16

**Authors:** Yen-Feng Lin, Chia-Yen Chen, Dost Öngür, Rebecca Betensky, Jordan W. Smoller, Deborah Blacker, Mei-Hua Hall

**Affiliations:** 1 Department of Epidemiology, Harvard T. H. Chan School of Public Health, Boston, MA USA; 2 Department of Psychiatry, Taipei City Psychiatric Center, Taipei City Hospital, Taipei, Taiwan; 3 Psychiatric and Neurodevelopmental Genetics Unit, Center for Human Genetic Research, Massachusetts General Hospital, Boston, MA USA; 4 Stanley Center for Psychiatric Research, Broad Institute of MIT and Harvard, Cambridge, MA USA; 5 Department of Psychiatry, Harvard Medical School, Boston, MA USA; 6 Psychotic Disorders Division, McLean Hospital, Belmont, MA USA; 7 Department of Biostatistics, Harvard T. H. Chan School of Public Health, Boston, MA USA; 8 Gerontology Research Unit, Massachusetts General Hospital, Boston, MA USA; 9 Psychosis Neurobiology Laboratory, McLean Hospital, Belmont, MA USA

## Abstract

Event-related potential (ERP) components have been used to assess cognitive functions in patients with psychotic illness. Evidence suggests that among patients with psychosis there is a distinct heritable neurophysiologic phenotypic subtype captured by impairments across a range of ERP measures. In this study, we investigated the genetic basis of this “globally impaired” ERP cluster and its relationship to psychosis and cognitive abilities. We applied *K*-means clustering to six ERP measures to re-derive the globally impaired (*n* = 60) and the non-globally impaired ERP clusters (*n* = 323) in a sample of cases with schizophrenia (SCZ = 136) or bipolar disorder (BPD = 121) and healthy controls (*n* = 126). We used genome-wide association study (GWAS) results for SCZ, BPD, college completion, and childhood intelligence as the discovery datasets to derive polygenic risk scores (PRS) in our study sample and tested their associations with globally impaired ERP. We conducted mediation analyses to estimate the proportion of each PRS effect on severity of psychotic symptoms that is mediated through membership in the globally impaired ERP. Individuals with globally impaired ERP had significantly higher PANSS-positive scores (β = 3.95, *P* = 0.005). The SCZ-PRS was nominally associated with globally impaired ERP (unadjusted *P* = 0.01; *R*^2^ = 3.07%). We also found a significant positive association between the college-PRS and globally impaired ERP (FDR-corrected *P* = 0.004; *R*^2^ = 6.15%). The effect of college-PRS on PANSS positivity was almost entirely (97.1%) mediated through globally impaired ERP. These results suggest that the globally impaired ERP phenotype may represent some aspects of brain physiology on the path between genetic influences on educational attainment and psychotic symptoms.

## Introduction

In recent years, traditional psychiatric diagnostic constructs have been increasingly challenged. This is particularly evident in psychotic spectrum disorders such as schizophrenia (SCZ), schizoaffective disorder (SA), and psychotic bipolar disorder (BPD). These disorders overlap substantially in symptoms, neurobiology, cognitive features, treatment response, and liability risk factors^1,2^. Moreover, large-scale genetic studies have consistently found overlap in susceptibility across BPD, SA, SCZ, and related phenotypes^3–5^. In line with shared genetic susceptibility, the endophenotype–biomarker literatures on BPD–SCZ indicate differences in degree, rather than differences in kind, across various domains of brain function, both in patients and in their clinically unaffected relatives^6–12^. These observations challenge the traditional dichotomous model of SCZ and BPD and support a dimensional approach to understanding how genetic and neurobiological underpinnings cut across diagnostic boundaries.

Auditory event-related potential (ERP) components—including P50 sensory gating, N1, P2, and P3—have been extensively investigated in the psychoses and are putative endophenotypes for psychotic spectrum disorders^7,13–17^. Each of these ERP components measures specific aspects of brain function and is reliably quantifiable across diverse clinical and laboratory settings^11,18^. P50 sensory gating probes inhibitory mechanisms thought to be crucial for protecting the brain from information overload^19^. Response to S1 stimulus assesses basic brain functions associated with auditory perception^20^. N1 ERP indexes sensory processing at the level of auditory cortex^17^. P2 and P3 components are associated with higher-order cognitive processes relevant to attention, working memory, and information processing speed^21,22^ (see Supplementary Information for details on ERPs). In twin analyses, we have shown that ERP phenotypes are heritable and genetically correlated with BPD and SCZ^8,9,15^.

We have also identified multivariate clusters of ERP phenotypes that appear to aggregate among individuals with psychotic BPD and SCZ, independent of diagnosis^23^. In that study, various domains of brain function, ranging from the early pre-attentive stage of information processing to higher complex cognitive processes (including P50 sensory gating, gamma band response, mismatch negativity, and the N1, P2, and P3 ERPs), were included to allow us to empirically derive homogenous subgroups based on these features. One of the clusters was termed “globally impaired” because this group of subjects exhibited functional abnormalities on all of these ERP measures. Such data-driven clustering holds promise for parsing the neurobiological and genetic heterogeneity of psychotic illness, and the analysis of phenotypes based on these clusters may enhance the power of genetic association analyses^24,25^. Importantly, the neuro-clusters identified in our study resembled the “Biotypes” recently reported by the Bipolar-Schizophrenia Network for Intermediate Phenotypes (BSNIP) consortium^10^, even though somewhat different biomarker panels were used in each study. Taken together, these results represent a diagnosis-free approach to integrate information across biomarkers, yielding neurobiologically distinct subgroups, and provide evidence supporting the potential role of neurobiological classification in differentiating individuals with psychotic disorders. The “globally impaired” ERP cluster identified in our prior work was found to be associated with psychotic illness and symptoms across diagnostic boundaries, but its genetic relationship to psychotic illness is unclear.

Findings from well-powered genome-wide association study (GWAS) indicate that psychiatric disorders are highly polygenic, reflecting the influence of thousands of common variants (single-nucleotide polymorphisms (SNPs)) of small effect. Although the individually modest effects of common variants make them uninformative as risk biomarkers, genome-wide polygenic risk scores (PRSs), which aggregate the effects of multiple SNPs from GWAS, can capture a substantial liability to disease risk^26^. The PRS for SCZ and BPD could be used to examine the degree to which multiple risk loci for psychotic illness overlap with those influencing the globally impaired ERP cluster, and such polygenic overlap could provide support for the globally impaired ERP as a putative endophenotype for psychotic illness.

ERP components have been used to assess cognitive functions in patients with psychotic illness. Although cognitive impairment is considered as a core feature of SCZ^27^ and psychotic BPD^28,29^, the relationship between cognitive performance and SCZ–BPD disorders is complex and controversial. Several epidemiological studies have reported low cognitive ability and poor school performance as risk factors for SCZ and BPD^30–32^. However, other studies have found a higher risk of developing psychotic illness among individuals with high levels of cognitive performance and creativity^33–36^. In addition, recent analysis of cross-trait genetic correlation found a positive genetic correlation between psychotic illness and higher educational attainment^37–40^, which has been used as a proxy for adolescent and young adult cognitive ability in genetic research^41,42^. PRSs for both SCZ and BPD were also reported to be highly positively associated with creativity and educational attainment^43^. Therefore, it is worthwhile to further examine the genetic relationship between cognitive ability and ERPs, an electrophysiological index of cognitive functions in psychotic illness.

In the present study, we first used a new sample with a similar panel of ERP phenotypes as those used in our previous study, performing *K*-means multivariate analyses to derive empirical clusters and see if we could replicate the previously derived neuro-clusters and the association between globally impaired ERP and psychotic symptoms^23^. We then constructed genome-wide PRSs for psychiatric and cognitive phenotypes, including SCZ, BPD, educational attainment, and childhood intelligence, to examine the shared genetic components between globally impaired neuro-cluster and each psychiatric or cognitive phenotype. Finally, we used a novel approach combining polygenic profiling and causal mediation methods to test the hypothesis that the “globally impaired” ERP profile represents an intermediate phenotype that mediates genetic influences on the symptomatology of SCZ and BPD. We performed causal mediation analyses to explore whether the “globally impaired profile” is a mediator between PRS and specific clinical features.

## Methods

### Study sample

The study sample consisted of 258 cases (SCZ = 136 and psychotic BPD = 122) and 125 healthy controls (prior to genetic quality control procedures). Cases were recruited from McLean Hospital, and healthy controls were recruited through local advertisements. All participants were assessed with the Structured Clinical Interview for DSM Disorders (SCID-I)^44^. All participants were of self-reported European ancestry, between 18 and 65 years of age, with no history of neurological disorders, no history of head injury, no substance abuse (except nicotine) or dependence in the preceding 12 months, normal hearing confirmed by audiometric testing, and normal intelligence based on the North American Adult Reading Test (NAART). All cases did not receive ECT treatment in the preceding 12 months, and were sufficiently stable to participate on an outpatient basis. All controls had no history of psychotic and mood disorders themselves or in a first-degree relative. Because of possible genetic overlap between psychosis and mood disorders, the healthy control group included only those free of mood disorders to optimize power to detect genetic risk factors.

This study was approved by the institutional review board at McLean Hospital. Written informed consent was obtained from all participants after fully explaining the aims and procedures of the studies.

### Clinical assessments

All participants completed the SCID-IV diagnostic interview, the Snaith–Hamilton Pleasure Scale (SHPS)^45^, and the Mood and Anxiety Symptom Questionnaire (MASQ)^46^. Demographic (age, sex, years of education, smoking status) and medication information were also obtained from participants. Treatment with antipsychotic medication was quantified in terms of chlorpromazine (CPZ) equivalents^47^. Among the 258 cases, 161 (SCZ *n* = 77, BPD *n* = 84) had data on Positive and Negative Syndrome Scale (PANSS) scores^48^, 243 (SCZ *n* = 121, BPD *n* = 122) had data on Young Mania Rating Scale (YMRS) scores^49^, and 138 (SCZ *n* = 66, BPD *n* = 77) had data on Multnomah Community Ability Scale (MCAS) scores^50^. There was no observed association between globally impaired ERP and missing data on each of the rating scales (all *P* > 0.20).

### Electrophysiological phenotypic measures

All participants completed the following tasks: an auditory dual-click paradigm^51^ and an auditory “oddball” paradigm^52^. We applied the same electroencephalogram (EEG) recording and processing procedures as described previously^15,23^. Briefly, EEG was recorded using the BioSemi Active Two system at a digitization rate of 512 Hz, with a bandpass of DC–104 Hz and a Common Mode Sense as the reference (PO2 site, parieto-occipital electrode 2) using an 18-channel electrode cap. Blinks and eye movements were monitored through electrodes placed on the left temple and above and below the left eye. The EEG data were re-referenced off-line to the averaged mastoid. Subjects were not allowed to smoke for a minimum of 40 min prior to the recordings. P50 sensory gating and response to S1 stimulus were elicited using the dual-click paradigm. The P50 sensory gating ERP was reported at the Cz site (central (C), midline of the head (z)) and calculated as a ratio (S2/S1) × 100, where higher ratios reflect more impairment. N1 and P2 amplitude ERPs were elicited by the response to the standard stimuli in the auditory Oddball paradigm and reported at the Cz site, whereas P3 amplitude and latency ERPs were elicited by the response to the target stimuli in the Oddball paradigm reported at the Pz site (parietal (P)) (see Supplementary Methods for detail).

### K-means cluster analyses

As in our previous study^23^, we included all participants (cases and healthy controls) in the analysis to empirically identify homogeneous subgroups of individuals who share similar neurophysiological profiles, regardless of diagnostic status. Individuals were clustered into three distinct subgroups using the *K*-means algorithm^53^ implemented in JMP (version 12.0, SAS Institute Inc.), according to six ERP measures: P50 sensory gating, amplitude of S1 response, N1 amplitude, P2 amplitude, P3 amplitude, and P3 latency. A globally impaired cluster, an intermediate cluster, and a high cognitive functioning cluster were empirically derived (Supplementary Table S2). The number of clusters was initially set at 3, based on our previous analysis^23^. We also applied a V-fold cross-validation method^54^ to a range of numbers of clusters (from 2 to 5) and identified 3 as the optimal value of *K* for *K*-means.

In our analyses, individuals in the globally impaired cluster were compared to those in the other two clusters (globally impaired (*n* = 60) vs. non-globally impaired (*n* = 323)). We treated the ERP clusters as categories based on our hypothesis that the globally impaired ERP, in particular, may be a useful phenotype for genetic studies.

### Genotyping and quality control

Genomic DNA from blood samples was extracted by standard procedures at the Massachusetts General Hospital Center for Genomic Medicine. Genotyping was performed at the Broad Institute using the Illumina Infinium OmniExpress array (Illumina Inc., San Diego, CA, USA). The quality control (QC) procedures have been described elsewhere^14^. Briefly, we excluded 9 individuals with discordant sex information, missing genotype rate >5% or heterozygosity rate >3 SD, shared inflammatory bowel disease >0.125, or non-European ancestry based on principal component analyses. We removed ~45,000 SNPs on the X or Y chromosome, minor allele frequency <0.05, call rate <98%, and *P* < 1 × 10E−6 for deviation from Hardy–Weinberg equilibrium. The QC steps were carried out with PLINK^55^ and resulted in a total of 374 subjects with genotype data on 664,907 autosomal SNPs.

We then performed genotype imputation, using the phased haplotypes from the 1000 Genomes Project dataset as the reference panel. Prephasing and imputation was done with SHAPEIT and IMPUTE2^56,57^. The imputation was performed with the default parameters of the software. The final imputed dataset consisted of 9.7 million autosomal SNPs.

### Statistical analyses

#### Phenotypic association analyses

*T*-tests, chi-square tests, or multivariable linear regression analyses were used (STATA version 12; Stata Corp., College Station, TX) to compare the demographic and clinical characteristics between the globally impaired ERP group and the non-globally impaired ERP group.

#### PRS association analyses

We used GWAS summary statistics for SCZ^58^ and BPD^59^ from the Psychiatric Genomics Consortium (PGC), educational attainment (college completion)^41^ from the Social Science Genetic Association Consortium (SSGAC), and childhood intelligence^60^ from the Childhood Intelligence Consortium (CHIC) as the discovery datasets to derive genome-wide PRSs^61^ for each of the above psychiatric or cognitive phenotypes in the study sample. The SCZ discovery sample consisted of 46 non-overlapping case–control samples (33,356 cases and 43,724 controls) and 3 family-based samples (1396 parent affected–offspring trios). The BPD discovery sample included 11 case–control samples (7481 cases and 9250 controls). The college completion discovery sample were combined from 42 GWAS samples (22,475 college and 78,594 non-college), and 95.8% of the individuals were older than 30 years. The childhood intelligence discovery sample consisted of 6 cohorts with a total of 12,411 children aged 6 to 18 years. All subjects in the discovery samples were of European ancestry. There were no overlapping individuals between these discovery samples and our study sample.

To account for only independent association signals from these discovery GWAS, we applied a linkage disequilibrium (LD) clumping procedure to each discovery dataset, in which we retained the SNP with smallest *P* value in each 250 kb window and removed all those in LD (*r*^2^ > 0.1) with this SNP. We also excluded the major histocompatibility complex region between 26 and 33 Mb on chromosome 6 when calculating the PRSs, because of the complex haplotype and LD structure in this region. For each psychiatric or cognitive phenotype, we used five different association *P* value thresholds (*P*_T_s)—0.001, 0.01, 0.05, 0.1, and 0.5—to select index SNPs from the clumped independent SNPs for calculating the PRSs. For each individual, we calculated the PRS for each psychiatric or cognitive phenotype by summing the risk allele counts of the index SNPs, weighted by the log of their association odds ratios (for SCZ, BPD, and college completion) or the beta coefficients (for childhood intelligence) estimated from the discovery GWAS results.

We used PRSice v1.23^62^ to calculate the PRSs and test the association between each PRS and the globally impaired ERP group. Associations were tested using logistic regression models including the top 3 principal components (PCs) of ancestry from the EIGENSTRAT analysis^63^ as covariates. We adjusted for the first 3 PCs because the 4th PC offers very little increase (<2%) in the total explained variance. Wald test *P* values and Nagelkerke’s *R*^2^s are reported. We performed the above PRS association analyses on the entire study sample and then repeated the same analyses on the case-only subsample. We used POLYGENESCORE software in R^64^ to calculate statistical power for the association between each PRS and the globally impaired ERP (see Supplementary Methods.)

### Causal mediation analyses

#### Relationship between PRS, globally impaired ERP, and PANSS-positive score

For each psychiatric or cognitive phenotype that gave evidence of PRS association with globally impaired ERP, we selected the PRS with a *P* value threshold that showed the strongest association, and examined its relationship with globally impaired ERP and PANSS-positive score in our study sample. We performed regression-based causal mediation analyses to examine whether globally impaired ERP might play a crucial mediating role in the polygenic effect on psychotic symptoms.

In these analyses we estimated the direct effect of each associated PRS (highest vs. lowest quartile) on the PANSS-positive score and the indirect effect mediated by globally impaired ERP (binary, globally impaired vs. non-globally impaired), adjusting for the top 3 PCs of ancestry, age, sex, daily CPZ equivalent dose of antipsychotics, and current smoking status at the time of EEG recording, which were potential exposure–mediator, exposure–outcome, or mediator–outcome confounders. The proportion mediated was obtained by dividing the estimated indirect effect by the estimated total effect, as an index of the degree of mediation. This method is based on the counterfactual framework for causal inference^65^, which is an extension of traditional regression-based mediation approaches^66^, allowing binary mediators and outcomes as well as exposure–mediator interactions^67^.

#### Relationship between PRS, diagnosis, and globally impaired ERP

Because PRS for any of the psychiatric or cognitive phenotypes may be associated with the diagnosis of psychotic illness^5,38^, it is possible that the observed relationship between a PRS and globally impaired ERP is a secondary consequence of the PRS effect on psychotic illness. To understand whether the effect of any associated PRS on globally impaired ERP is mediated through “case vs. control status” (i.e., presence vs. absence of psychotic illness) or through one specific major mental illness (SCZ vs. BPD among cases), we also performed mediation analyses to understand the relationships between PRS, diagnosis, and Globally impaired ERP (see Supplementary Methods).

#### Sensitivity analyses

Finally, we conducted sensitivity analyses to evaluate the robustness of the above mediation analyses to unmeasured confounding (see Supplementary Methods). All mediation analyses were performed using the PARAMED module in STATA^68^. We used bootstrap procedures with 200 replications to compute a 95% bias-corrected bootstrap confidence interval (95% BCCI) for the direct and indirect effects.

## Results

### Phenotypic associations with globally impaired ERP

The demographic and clinical characteristics of the globally impaired ERP and the non-globally impaired ERP are presented in Table 1. In the analysis of all participants, the globally impaired cluster consisted of primarily SCZ or BPD cases (91.7%, which included 48.3% of SCZ cases, 43.3% of BPD cases, vs. 8.3% of controls). The small difference between the proportion of the two disorder groups classified as either globally impaired was not significant. Individuals in the globally impaired cluster were significantly older (*P* = 0.007) and were more likely to be current smokers (*P* = 0.005).

**Table 1:** Demographic and clinical characteristics of globally impaired and non-globally impaired clusters

Phenotype characteristics	All subjects		
	Globally impaired ERP	Non-globally impaired ERP	*P* value
Diagnosis			X^2^ = 19.11, *P* = **7.07E**−**05**
SCZ, *N* (%)	29 (48.3)	107 (33.1)	
BPD, *N* (%)	26 (43.3)	96 (29.7)	
Unaffected, *N*(%)	5 (8.3)	120 (37.2)	
Sex			X^2^ = 0.18, *P* = 0.68
Female, *N* (%)	30 (50.0)	171 (52.9)	
Age (years), mean (SD)	43.58 (14.68)	38.41 (13.25)	*t*-test, *P* = **0.007**
Education (years), mean (SD)	14.58 (2.22)	14.98 (2.27)	*t*-test, *P* = 0.22
Current smoker, *N* (%)	24 (42.1)	77 (24.2)	X^2^ = 7.86, *P* = **0.005**
MASQ total, mean (SD)	130.53 (37.62)	121.27 (36.70)	*t*-test, *P* = 0.11 MLR, *P* = 0.62
SHPS, mean (SD)	1.84 (2.87)	1.09 (1.92)	*t*-test, *P* = **0.02** MLR, *P* = 0.29
	**Cases with SCZ or BPD**		
Diagnosis			X^2^ < 0.0001, *P* = 1.00
SCZ, *N* (%)	29 (52.7)	107 (52.7)	
BPD, *N* (%)	26 (47.3)	96 (47.3)	
Sex			X^2^ = 2.47, *P* = 0.12
Female, *N* (%)	26 (47.3)	120 (59.1)	
Age (years), mean (SD)	45.02 (14.39)	41.38 (12.68)	*t*-test, *P* = 0.07
Education (years), mean (SD)	14.38 (2.2)	14.62 (2.2)	*t*-test, *P* = 0.50
Current smoker, *N* (%)	24 (46.2)	69 (34.3)	X^2^ = 2.48, *P* = 0.12
Age of onset (years), mean (SD)	22.35 (8.4)	22.87 (8.3)	*t*-test, *P* = 0.70
CPZ equivalent dosage (mg), mean (SD)	286.06 (336.70)	376.62 (508.10)	*t*-test, *P* = 0.24
PANSS positive total, mean (SD)	19.88 (7.49)	16.13 (6.98)	*t*-test, *P* = **0.007** MLR, ***P*** = 0.005
PANSS negative total, mean (SD)	13.18 (7.46)	12.22 (5.64)	*t*-test, *P* = 0.42 MLR, *P* = 0.31
PANSS general total, mean (SD)	32.82 (8.61)	30.28 (9.56)	*t-*test, *P* = 0.17 MLR, *P* = 0.11
MCAS total, mean (SD)	44.54 (8.19)	46.83 (5.71)	*t*-test, *P* = 0.09 MLR, *P* = 0.28
YMRS total, mean (SD)	7.81 (12.22)	8.64 (11.19)	*t*-test, *P* = 0.64 MLR, *P* = 0.91
MASQ total, mean (SD)	134.93 (36.9)	136.49 (38.6)	*t*-test, *P* = 0.81 MLR, *P* = 0.83
SHPS, mean (SD)	2.02 (2.96)	1.65 (2.3)	*t*-test, *P* = 0.39 MLR, *P* = 0.31

*X*^2^ is chi-square statistic

*t*-test is two-sample t-test for equal means

MLR is multivariable linear regression for the association between clinical assessments and globally impaired ERP, adjusting for: (1) age, sex, case–control status, and current smoking status for all subjects; or (2) age, sex, daily chlorpromazine equivalent dose of antipsychotics, and current smoking status for cases with SCZ or BPD

All bold values are significant at *P* < 0.05

All tests are two sided

In the analysis restricted to cases only, there was no significant difference in age or other demographic variables between the two ERP clusters. However, SCZ/BPD cases in the globally impaired cluster had significantly higher PANSS-positive scores than those in the non-globally impaired cluster (mean (SD): 19.88 (7.49) vs. 16.13 (6.98); *P* = 0.007), and these differences persisted after adjusting for age, sex, daily chlorpromazine equivalent dose of antipsychotics, and smoking status at the time of EEG recording (multivariable linear regression: β = 3.95, *P* = 0.005).

Supplementary Table S1 presents demographic and clinical information for the study sample by diagnostic group.

### PRS association analyses

**Fig. 1:**
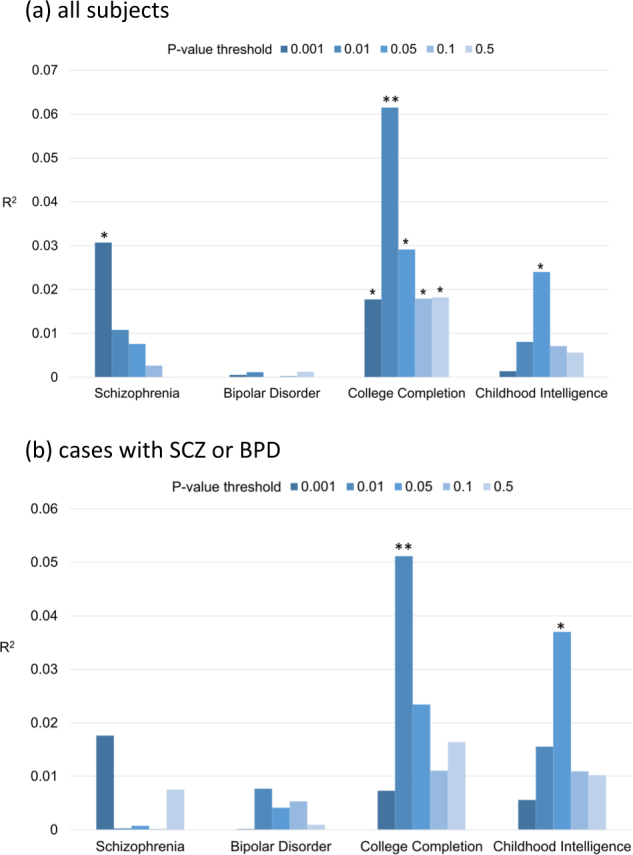
**Pair-wise polygenic association analyses between globally impaired ERP and PRS for each psychiatric or cognitive phenotype for: (a) all subjects and (b) cases with SCZ or BPD.** We derived PRS for schizophrenia, bipolar disorder, college completion, and childhood intelligence from each of the discovery samples with five different *P* value thresholds (PTs used to select training set SNPs: 0.001, 0.01, 0.05, 0.1, and 0.5; shown with different colors) and apply them to globally impaired ERP in **(a)** the entire sample and **(b)** those affected by SCZ or BPD. Each pair is shown on the *x*-axis and the proportion of variance explained for globally impaired ERP (estimated via Nagelkerke’s pseudo-*R*²) on the *y*-axis. *Unadjusted *P* value < 0.05; **FDR-corrected *P* value < 0.05 (the total number of testing for multiple comparisons *n* = 20)

Results of PRS associations between the globally impaired ERP cluster and SCZ-PRS, BPD-PRS, college-PRS, and childhood intelligence-PRS including all subjects are presented in Fig. 1a and Supplementary Table S3a. Results restricted to cases only are presented in Fig. 1b and Supplementary Table S3b. In the full sample analyses, the SCZ-PRS with a *P* value threshold of 0.001 (SCZ-PRS_PT=0.001_) was significantly positively associated with risk of globally impaired ERP (unadjusted *P* = 0.01; *R*^2^ = 3.07%). This association approached significance (false discovery rate (FDR)-corrected *P* = 0.06) even after correcting for multiple testing (*n* = 20) by the FDR *q*-value method^69,70^. In the analyses restricted to cases only, results were not significant but were in the same direction (unadjusted *P* = 0.09, *R*^2^ = 1.76%; FDR-corrected *P* = 0.17). For the BPD-PRS, no significant associations were found with the globally impaired ERP cluster in either the whole sample or the case-only subsample.

In the full sample analyses, we found a significant positive association between the college-PRS and the globally impaired cluster across all five *P* value thresholds (unadjusted *P* values range from 2.95E−04 to 0.05, see Supplementary Table S3a), such that alleles associated with higher educational attainment were associated with being in the globally impaired cluster. After multiple testing correction, this association remained significant for the college-PRS with a *P*_T_ = 0.01 (college-PRS_PT=0.01_, FDR-corrected *P* = 0.004; *R*^2^ = 6.15%). We also observed a nominally positive association between the childhood intelligence-PRS with *P*_T_ = 0.05 and the globally impaired cluster (unadjusted *P* = 0.02, *R*^2^ = 2.40%; FDR-corrected *P* = 0.08). In the case-only subsample, we again found a significant positive association between the college-PRS_PT=0.01_ and globally impaired ERP membership (unadjusted *P* = 0.004; FDR-corrected *P* = 0.04; *R*^2^ = 5.11%; see Supplementary Table S3b) and a positive association between the childhood intelligence-PRS_PT=0.05_ and globally impaired ERP (unadjusted *P* = 0.01; FDR-corrected *P* = 0.06; *R*^2^ = 3.70%).

Results of the power analysis for detecting the association between each PRS and the globally impaired ERP are presented in Supplementary Tables S4a-4d. These results may mitigate concerns that the differential associations across PRS are due to difference in the power of GWAS for each phenotype.

### Mediation analyses

#### Relationship between SCZ-PRS, globally impaired ERP, and PANSS-positive score

**Fig. 2:**
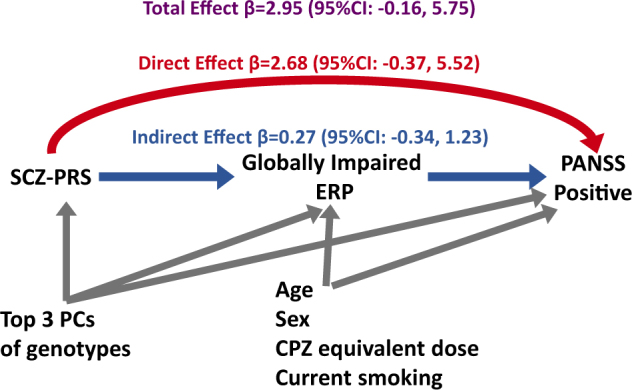
**Causal relationship between SCZ-PRS, globally impaired ERP, and PANSS-positive score for patients**

As noted above, the SCZ-PRS with a *P* value threshold of 0.001 (SCZ-PRS_PT=0.001_) was nominally associated with the globally impaired cluster, and this association approached significance after correcting for multiple testing. Because of the observed association between globally impaired ERP and PANSS-positive scores among cases, we further examined whether SCZ-PRS_PT=0.001_ is also associated with PANSS-positive score and whether this relationship is mediated by globally impaired ERP (Fig. 2). The estimated direct and indirect effects betas were 2.68 (95% BCCI: −0.37, 5.52) and 0.27 (95% BCCI: −0.34, 1.23), respectively. The proportion of estimated mediating effect of globally impaired ERP on the total effect of SCZ-PRS on PANSS-positive score was small (9.1%). Adding an exposure–mediator interaction term did not substantially change the effect estimates (direct effect β = 2.30 (95% BCCI: −0.82, 5.18); indirect effect β = 0.44 (95% BCCI: −0.63, 1.92)). The minimal effect of including the interaction term suggests that exposure–mediator interaction did not appear to be substantial^71^.

#### Relationship between college-PRS, globally impaired ERP, and PANSS-positive score

**Fig. 3:**
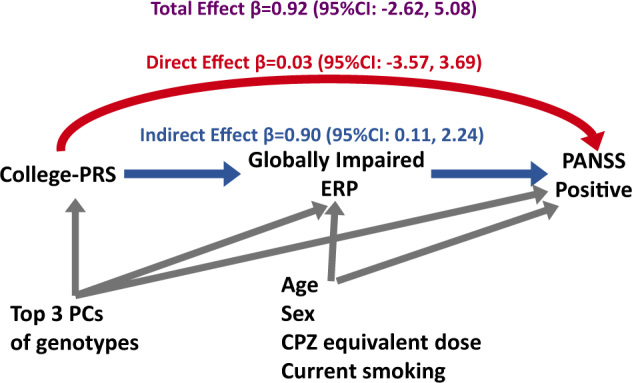
**Causal relationship between college-PRS, globally impaired ERP, and PANSS-positive score for patients**

We found a significant positive association, even after multiple testing correction, between the globally impaired cluster and the college-PRS at the *P* value threshold of 0.01. We further examined whether college-PRS_PT=0.01_ is also associated with PANSS-positive score and whether this relationship is mediated by globally impaired ERP. Results of the analysis with globally impaired ERP cluster as a mediator between college-PRS_PT=0.01_ and PANSS-positive symptoms are presented in Fig. 3. The total effect of the college-PRS_PT=0.01_ on PANSS-positive score was estimated as 0.92 (95% BCCI: −2.62, 5.08). The direct effect was estimated to be β = 0.03 (95% BCCI: −3.57, 3.69) and the indirect effect mediated through globally impaired ERP was estimated to be β = 0.90 (95% BCCI: 0.11, 2.24) (Fig. 3). The proportion of mediating effect from college-PRS through globally impaired ERP to PANSS positive was estimated at 97.1%. These results suggest that the effect of the college-PRS_PT=0.01_ on PANSS-positive score was almost entirely mediated through globally impaired ERP. Adding an exposure–mediator interaction term did not substantially change the effect estimates (direct effect β = −0.22 (95% BCCI: −3.97, 3.59); indirect effect β = 1.12 (95% BCCI: 0.06, 3.34)).

#### Relationship between PRS, diagnosis, and globally impaired ERP

We also examined whether the effect of any associated PRS on globally impaired ERP is mediated through “case vs. control status” or through one specific major mental illness among cases (see Supplementary Results). Nearly one-third (30.9%) of the total effect of SCZ-PRS_PT=0.001_ on globally impaired ERP was mediated by the presence of psychotic illness (Figure S1a). Among cases, the proportion of estimated mediating effect of “SCZ vs. BPD” on the total effect of SCZ-PRS_PT=0.001_ on globally impaired ERP was very close to zero (Figure S1b). A small proportion (11%) of the total effect of college-PRS_PT=0.01_ on globally impaired ERP was mediated by the presence of psychotic illness (Figure S2a). For cases with psychotic illness, the mediating effect due to having a specific diagnosis of SCZ or BPD was estimated to be zero (Figure S2b).

#### Sensitivity analyses of unmeasured confounding

Sensitivity analyses of unmeasured confounding suggest that even in the presence of strong unmeasured confounding, results of the above mediation analyses would not substantially change (see Supplementary Results and Table S5-S10). In the mediation analysis with college-PRS as the exposure, globally impaired ERP profile as the mediator, and PANSS-positive score as the outcome, existence of unmeasured confounding would likely lead to overestimation of the indirect effect and underestimation of the direct effect. Nonetheless, the estimated indirect effect remained significant after controlling for a strong hypothetical confounder with correlations of 0.3 with both mediator and outcome, and the proportion mediated of 66.5% supported our conclusion that the majority of the effect of college-PRS_PT=0.01_ on PANSS-positive score was indirect.

## Discussion

In the present study, we successfully replicated the clustering of ERP components in an independent sample, including a globally impaired ERP cluster (defined as having abnormalities in all six ERP measures, including P50 sensory gating, amplitude of S1 response, N1 amplitude, P2 amplitude, P3 amplitude, and P3 latency). We also replicated our previous findings that individuals in the globally impaired cluster exhibited greater psychotic symptom severity than individuals in other clusters (Table 1)^23^. Our results showed that the globally impaired ERP phenotype was associated with polygenic influences on educational attainment and, to a lesser extent, schizophrenia. We also observed a positive association between education PRS and positive symptoms that was almost entirely mediated by effects on the globally impaired ERP phenotype.

Our results demonstrate possible polygenic pleiotropy between SCZ and globally impaired ERP. We found that higher SCZ polygenic risk was marginally associated (unadjusted *P* value = 0.01, FDR-corrected *P* = 0.06) with being in the globally impaired ERP cluster. However, there was no observed association between BPD-PRS and the globally impaired ERP cluster.

Although globally impaired ERP was associated with both SCZ-PRS and PANSS-positive symptoms score, showing potential to serve as an endophenotype for schizophrenia, only a small proportion (9.1%) of the effect of SCZ-PRS on PANSS-positive score was mediated by globally impaired ERP, suggesting that ERP cluster may not be an ideal intermediate phenotype between SCZ-related genetic variants and positive psychotic symptoms. In addition, we found that the relationship between SCZ-PRS and globally impaired ERP was significantly mediated by the presence of psychotic illness (i.e., case vs. control status) (see Supplementary Figure S1a), implying that the observed association between SCZ-PRS and globally impaired ERP may be only secondary to the effects of SCZ-associated SNPs on the presence of psychotic illness.

The evidence for polygenic overlap was strongest for college completion and globally impaired ERP. We found significant positive PRS correlations between greater college-PRS (i.e., greater polygenic loading for higher education) and the globally impaired cluster across all five *P* value thresholds (Supplementary Table S3a), with the strongest signal at the PRS *P* value threshold of 0.01, explaining 6% of the variance in the globally impaired ERP in the full sample (*n* = 383). A similar pattern of genetic overlap was also observed between greater childhood intelligence-PRS and being in the globally impaired cluster. These results were unexpected, as cognitive impairment is common among patients with SCZ and BPD and epidemiological studies have indicated that poor school performance and low cognitive ability are risk factors for SCZ and BPD^30–32^. However, our results are compatible with findings for BPD from the Swedish National School Register of over 900,000 individuals showing that those with excellent school performance had a nearly fourfold increased risk of later BPD compared with those with average grades^36^. Our results are also consistent with recent findings examining genetic overlap between psychiatric diseases and cognitive ability. Studies employing an LD score regression approach to estimate cross-trait genetic correlations found positive genetic correlations between BPD/SCZ risk and educational attainment^38,40^. One possible explanation for the LD score regression results is “case ascertainment bias,” such that patients from more educated families were more likely to participate in research. However, in our study, we avoided such case ascertainment bias by using an objective physiological phenotype, which was not phenotypically associated with years of education, and found a significant positive genetic correlation between this psychosis-related trait and higher educational attainment. Further research is needed to replicate and explain the counterintuitive genetic correlation between higher educational attainment and globally impaired ERP.

We found that the effect of the college-PRS_PT=0.01_ on PANSS-positive score was almost entirely mediated through globally impaired ERP membership (Fig. 3). It has been suggested that a dimensional classification of psychopathology among patients with SCZ and BPD can better reflect the underlying genetic variation;^2^ therefore, PANSS scores have been used in genetic research to identify the genetic underpinning of specific symptom dimensions of psychotic illness^72^. However, the major disadvantage of using specific symptom-domain scores (e.g., PANSS scores) as the phenotype is that they are very likely to be influenced by treatment, stage of illness, and other environmental factors. Globally impaired ERP as an intermediate phenotype of positive symptoms may be less likely to be influenced by clinical or environmental factors. The nearly complete mediation of the association between college-PRS and PANSS positive by globally impaired ERP implies that the globally impaired ERP may represent some aspects of brain physiology linking higher education-associated alleles and positive psychotic symptoms. By contrast, only a small proportion of the relationship between SCZ-PRS and PANSS-positive score was mediated by globally impaired ERP. It is possible that SNPs affecting positive symptom severity partially overlap with both SCZ-associated and education-associated SNPs, and globally impaired ERP may capture the component of positive symptoms that is genetically correlated with educational attainment. Thus, globally impaired ERP may help stratify the genetic components of psychotic symptoms (see Supplementary Figure S5).

The present study has several limitations. First, the PRS approach assumes a linear additive model and does not consider gene–gene interactions that may contribute to the underlying genetic architecture of the phenotypes of interest. Second, the effect estimates from the mediation analyses might be biased due to violation of the unmeasured confounding assumption^67^. However, our sensitivity analyses suggest that even with the existence of a strong unmeasured confounder for the mediator–outcome relationship, the results of mediation analyses remained robust. Third, our analyses were restricted to individuals of European ancestry, thus limiting the generalizability of the findings to other ethnic populations. Future research should include a broader range of ethnic populations. Finally, although the causal mediation relationship identified by a statistical approach may imply a mechanistic causality, the true mechanisms governing the processes from exposure to outcome can only be understood by considering the sufficient cause model (i.e., the identification of a set of minimal conditions that inevitably produce outcome). To look into the black box of causal mechanisms, closer observations, more detailed and extensive data, and more scientific knowledge will be needed.

## Conclusion

This is the first study, to our knowledge, to demonstrate a potential link between genetic risk scores, ERP phenotype, and positive psychotic symptoms. The results also support prior evidence that college education, a proxy for adolescent and young adult cognitive ability, is genetically correlated with psychotic illness, and suggest a potential physiological role for the multivariate ERP profile in the genetic link between cognitive ability and psychotic symptoms.

## Electronic supplementary material

Supplemental Materials
