# Supplementary material for: Polygenic pleiotropy and potential causal relationships between educational attainment, neurobiological profile, and positive psychotic symptoms
Source: Transl Psychiatry. 2018 May 16;8:97. doi: 10.1038/s41398-018-0144-4 (PMC5954124; doi:10.1038/s41398-018-0144-4)
Supplement: Supplementary file 1 — Supplemental Materials [file 41398_2018_144_MOESM1_ESM.docx]

**Polygenic Pleiotropy and Potential Causal Relationships between Educational Attainment, Neurobiological Profile, and Positive Psychotic Symptoms**

***Supplementary Information***

**SUPPLEMENTARY METHODS**

***Neurophysiological Recordings and Signal Processing***

***Event related potential (ERP)***

An ERP is a measured brain electrophysiological response to a specific sensory, cognitive, or motor event. ERPs can be reliably measured using electroencephalography (EEG), a procedure that records electrical activity of the brain over time using non-invasive electrodes placed along the scalp.

***Dual-Click Paradigm***

*P50 Sensory Gating.* The P50 sensory gating is the brain’s suppression of an evoked response to a brief stimulus, usually an auditory click, occurring approximately 50 milliseconds (ms) after receiving the stimulus.

In our study, the P50 sensory gating ERP was elicited using the dual-Click paradigm (160 pairs of identical click stimuli, 5-ms duration; 2-ms rise/fall; 500-ms inter-click interval; 10-s inter-trial interval). Signal processing was performed off-line using NEUROSCAN software (4.3).^1, 2^EEG signals were segmented (–100 to 400 ms), filtered (1-Hz high-pass filter), baseline corrected, and artifact rejected if activity exceeding 50 μV between 0 and 75 ms post-stimulus. S1 and S2 waveforms were averaged, digitally filtered (10-Hz high pass), and smoothed. P50 sensory gating ERP are reported at the Cz site and calculated as a ratio (S2/S1)×100. A higher ratio reflects more impairment. For the S1 response, the most prominent peak 40–80 ms post-stimulus was selected as the P50 peak. The preceding negative trough was used to calculate the amplitude. For the S2 response, the positive peak with the latency closest to that of the conditioning P50 peak was selected, and its amplitude was determined as for the S1 wave. P50 sensory gating was calculated as (S2/S1)×100.^1, 2^

***Oddball Paradigm***

*P300 (P3) ERP components.* The P3 wave is an endogenous ERP component elicited in the process of decision making. When recorded by electroencephalography (EEG), it surfaces as a positive deflection in voltage with a latency (delay between stimulus and response) of roughly 250 to 500 ms. The signal is typically measured most strongly by the electrodes covering the parietal lobe. It reflects processes involved in stimulus evaluation or categorization. The P3 wave is usually elicited using the oddball paradigm.

In our study, P3 amplitude and latency ERPs were elicited by the auditory Oddball paradigm (400 binaural tones; 50-msec duration, 5 ms rise/fall times; 15% 1500 Hz target tones; 85% 1000 Hz standard tones). All participants had >90% accuracy. Signal processing was performed off-line using Brain Vision Analyzer software. EEG signals were first re-referenced to linked mastoids and zero phase-shift digital low-pass filtered at 8.5Hz (24 dB/Oct). Eye-blink artifacts were corrected by using the method of Gratton et al..^3^ The EEG data were segmented into epochs from -100 to 1000 ms relative to stimulus onset and baseline corrected using the 100-ms pre-stimulus interval. Epochs containing artifact >100 μV were removed. Separate average waves for target and standard tones were calculated. P300 amplitude and latency components were measured from the average wave for target tones at the Pz site between 280 and 650 ms.^4, 5^

*N1P2 ERP components.* The N1 ERP component is a large, negative-going evoked potential. It peaks in adults between 80 and 120 milliseconds after the onset of a stimulus, and distributed mostly over the fronto-central region. It is elicited by any unpredictable stimulus in the absence of task demands. It is often referred to with the following P2 wave as the "N1-P2" complex. The P2 ERP component is a positive going electrical potential that peaks at about 200 ms (between about 150 and 275 ms) after the onset of external stimulus.

In our study, EEG data were digital low-pass filtered at 20Hz (24 dB/Oct), baseline corrected, eye-blink corrected using ^3^, and artifact rejected if activity exceeding >100 μV. Peak N1 amplitude was automatically detected as the most negative point from 50 to 200 ms at Cz. Peak P2 amplitude was automatically detected as the most positive point from 150 to 300 ms at Cz.^6-8^

***Power Calculation for the association between PRS and globally impaired ERP***

We used POLYGENESCORE software in R^9^ to calculate statistical power for the association between each PRS and globally impaired ERP. With known sample sizes (globally impaired/ non-globally impaired: 60/323 in the study sample; case/control: 34,752/46,516 in the SCZ discovery sample, 7,481/9,250 in the BPD discovery sample, and 22,475/ 78,594 in the college education discovery sample; and 12,411 in the childhood intelligence discovery sample), we calculated the power for detecting the PRS association when the genetic correlation between globally impaired and each psychiatric or cognitive phenotype is 0.1, 0.3, or 0.5, under the following assumptions:

(1) The prevalence of globally impaired is 0.04, same as the proportion of globally impaired ERP in healthy individuals in our study sample. The prevalence of both SCZ and BPD is 0.01. The prevalence of college completion is 0.22, same as the proportion of college completion in the college education discovery sample.

(2) The SNP-based heritability of SCZ, BPD, college completion, and childhood intelligence is 0.4541, 0.432, 0.0791, and 0.2735, respectively, according to LD score regression analysis results reported on the LD Hub website (<http://ldsc.broadinstitute.org/lookup/>).

(3) The SNP-based heritability of globally impaired ERP is assumed to be 0.1, 0.3, or 0.5.

(4) The number of independent SNPs in the gene score is assumed to be 1000 or 10000.

(5) The Type-I error of the test for association between the PRS and globally impaired in the target sample is 0.05.

The results of the power analyses are shown in Tables S4a-d.

***Supplementary Mediation Analyses***

**Relationship between** **PRS, Diagnosis, and Globally Impaired ERP**

For each psychiatric or cognitive phenotype that gave evidence of PRS association with globally impaired ERP, we selected the PRS with a P-value threshold that showed the highest association, and examined its relationship with the target phenotypes in our study sample. Because PRS for any of the psychiatric or cognitive phenotypes may be associated with the diagnosis of psychotic illness,^10, 11^ it is possible that the observed relationship between a PRS and globally impaired ERP is a secondary consequence of the PRS effect on psychotic illness. To understand whether the effect of any associated PRS on globally impaired ERP is mediated through “case vs. control status” (i.e., presence vs. absence of psychotic illness) or through one specific major mental illness (SCZ vs. BPD among cases), we performed a regression-based causal mediation analysis to decompose the total effect of each PRS on globally impaired into direct and indirect effects, adjusting for potential confounders.

In the first set of mediation analyses, each associated PRS exposure was categorized into quartiles, the potential mediator “case vs. control status” was binary, and the outcome “globally impaired ERP” was treated as a binary variable (globally impaired vs. non-globally impaired). The total effect of each PRS on globally impaired ERP was decomposed into direct and indirect (mediated) effects. These effects were estimated as the odds ratio (OR) for globally impaired comparing the highest quartile to the lowest quartile of the PRS, adjusting for age, sex, and the top 3 PCs of ancestry, which were potential exposure-mediator or exposure-outcome confounders. In the second set of mediation analyses, we performed the same analyses as above in cases, but replaced the mediator variable with diagnosis (SCZ vs. BPD), adjusting for age, sex, and the top 3 PCs of ancestry. We estimated the proportion mediated for each of the above on the log odds scale by dividing the log of the estimated indirect effect OR by the log of the estimated total effect OR (as an index of the degree of mediation).

***Sensitivity Analyses of Unmeasured Confounding***

The counterfactual-based mediation analysis assumes no unmeasured confounding for the (1) exposure-mediator, (2) exposure-outcome, and (3) mediator-outcome relationships.^12^ In our mediation analyses with PRS as the exposures, assumptions (1) and (2) were probably plausible, since we had adjusted for the top principal components (PCs) of genotypes to address possible population stratification. However, the assumption of no unmeasured confounding might be less plausible for the (3) mediator-outcome relationship, and the effect estimates would probably be biased.

In order to evaluate the robustness of the mediation analyses to unmeasured confounding between mediator and outcome, we conducted sensitivity analyses to calculate how much direct and indirect effect estimates would be expected to change under different degrees of mediator-outcome confounding. Specifically, given a hypothetical unmeasured confounder of the mediator-outcome relationship, U, with particular correlations with the mediator and the outcome, we would like to know, if we were able to also adjust for U, what the direct and indirect effect estimates would be.

For each mediation analysis, we generated four standard normal variables (i.e., mean=0 and variance=1), with particular correlations with the mediator and the outcome, as hypothetical confounders (Table S5-10). The first hypothetical confounder has correlation of 0.1 with the mediator and correlation of 0.1 with the outcome. The second hypothetical confounder has correlation of 0.3 with the mediator and correlation of 0.1 with the outcome. The third hypothetical confounder has correlation of 0.1 with the mediator and correlation of 0.3 with the outcome. The fourth hypothetical confounder has correlation of 0.3 with the mediator and correlation of 0.3 with the outcome. We compared the direct and indirect effect estimates before and after adjusting for each of these hypothetical confounders, to assess the potential impact of unmeasured confounding on each mediation analysis.

***GWAS of globally impaired ERP***

We also performed a preliminary GWAS for globally impairer ERP (globally impaired vs. non-globally impaired). With such a small sample size and lack of replication, we are aware that the GWAS results may not be reliable. We did this just to make the best use of our data and hope to contribute to future research.

We tested each genotyped and imputed SNP for association with globally impaired ERP group in the form of logistic regression assuming an underlying additive model in PLINK.^13^ We included the top 3 PCs from the EIGENSTRAT analysis^14^ as covariates. We obtained an estimated odds ratio (OR) and a P-value for the association test for each SNP.

**SUPPLEMENTARY RESULTS**

***Supplementary Mediation Analyses***

**Relationship between SCZ-PRS, Diagnosis, and Globally Impaired ERP**

The SCZ-PRS with a P-value threshold of 0.001 (SCZ-PRS_PT=0.001_) was significantly associated with the globally impaired cluster, and this association approached significance after correcting for multiple testing. Thus, as described in Supplementary Methods, we then conducted causal mediation analyses to determine whether the effect of SCZ-PRS_PT=0.001_ on globally impaired ERP was mediated by the presence of SCZ and BPD (together or individually). The results examining whether case vs. control status mediates the relationship between SCZ-PRS_PT=0.001_ and globally impaired ERP are presented in Figure S1a. The estimated direct effect OR was 1.76 (95% BCCI: 0.72, 3.80). The indirect effect OR was 1.29 (95% BCCI: 1.12, 1.54), significantly greater than 1. Nearly one-third (30.9%) of the total effect of SCZ-PRS_PT=0.001_ on globally impaired ERP was mediated by the presence of psychotic illness. Adding an exposure-mediator interaction term resulted in a minimal change in the effect estimates (direct effect OR =-1.67 [95% BCCI: 0.77, 3.62]; indirect effect OR=1.36 [95% BCCI: 1.12, 1.65]).

Figure S1b presents the results examining in the case only sample whether specific diagnosis (SCZ vs. BPD) mediates the relationship between SCZ-PRS_PT=0.001_ and globally impaired ERP. The estimated direct and indirect effects ORs were 2.28 (95% BCCI: 0.94, 6.08) and 1.00 (95% BCCI: 0.94, 1.10), respectively. The proportion of estimated mediating effect of “SCZ vs. BPD among cases” on the total effect of SCZ-PRS_PT=0.001_ on globally impaired ERP was very close to zero (0.2%).

**Relationship between college-PRS, Diagnosis, and Globally Impaired ERP**

We found a significant positive association, even after multiple testing correction, between college-PRS (at P_T_ = 0.01) and the globally impaired cluster. Since patients with SCZ or BPD were more likely to have globally impaired ERP, it is possible that the observed association between the college-PRS and globally impaired ERP could be partly explained by the presence of psychotic illness. We therefore performed two causal mediation analyses to understand whether the effect of college-PRS_PT=0.01_ on globally impaired ERP was mediated by diagnostic status. The results examining whether the effect of college-PRS _PT=0.01_ on globally impaired ERP was mediated by case vs. control status are presented in Figure S2a. The estimated direct effect OR was 4.09 (95% BCCI: 1.60, 10.59), while the estimated indirect effect OR mediated by case vs. control status was 1.19 (95% BCCI: 1.00, 1.46) (Figure 2a). When an interaction between college-PRS _PT=0.01_ and case vs. control status was included in the regression model, the estimated direct and indirect effect ORs were 4.12 (95% BCCI: 1.66, 10.25) and 1.16 (95% BCCI: 0.98, 1.38), respectively. The minimal effect of including the interaction term suggests that exposure-mediator interaction did not appear to be substantial ^12^. Overall, then, the effect of college-PRS _PT=0.01_ on globally impaired ERP appeared to be primarily explained by the direct (non-mediated) relationship, whereas the proportion of estimated mediating effect of psychotic illness on the total effect was small (11.1%).

The results examining whether specific diagnosis (SCZ vs. BPD among cases) mediates the relationship between college-PRS_PT=0.01_ and globally impaired ERP are presented in Figure S2b. The estimated direct and indirect effects ORs were 3.94 (95% BCCI: 1.47, 10.71) and 1.00 (95% BCCI: 0.93, 1.08), respectively. The mediating effect due to diagnosis was estimated to be zero, indicating that for cases with psychotic illness, the effect of the college-PRS_PT=0.01_ on globally impaired ERP is not mediated by having a diagnosis SCZ or BPD *per se*.

In the full sample, the effect of college-PRS on globally impaired ERP was only modestly (11.1%) mediated by case vs. control status (combining SCZ and BPD cases; Figure S2a). While college-PRS_PT=0.01_ was associated with globally impaired ERP, it was not associated with psychotic illness. One possible explanation is that the diagnosis of psychotic illness is heterogeneous, and the ERP phenotype captures the component of psychosis that is correlated with education-associated genes. If we hypothesize that there is some genetic overlap between higher education and psychotic illness, the ERP phenotype may be a better alternative phenotype than traditional diagnosis to detect such genetic overlap. Among cases with psychotic illness, the effect of college-PRS_PT=0.01_ on globally impaired ERP did not appear to be mediated by the specific diagnosis (SCZ vs. BPD) (Figure S2b), implying that the ERP phenotype may potentially identify genetically relevant groups independent of the diagnostic boundary between SCZ and BPD.

***Sensitivity Analyses of Unmeasured Confounding***

**SCZ-PRS as the exposure:**

Sensitivity analyses of unmeasured confounding found similar results after adjusting for hypothetical confounders in all three mediation analyses with SCZ-PRS as the exposure (Table S5-7).

In the analysis on patients with SCZ-PRS as the exposure, globally impaired ERP as the mediator, and PANSS positive score as the outcome reported in the main text, the estimated direct and indirect effects odds ratios (95%CI) before adjusting for the hypothetical unmeasured confounder were 2.68 (-0.37, 5.52) and 0.27 (-0.34, 1.23), respectively. The effect estimates after adjusting for each hypothetical confounder U are shown in Table S5. Under adjustment of a strong hypothetical confounder with correlations of 0.3 with both mediator and outcome, the estimated direct and indirect effects odds ratios (95%CI) were 2.49 (-0.21, 5.77) and 0.15 (-0.18, 1.04), respectively, which were very close to the unadjusted effect estimates.

In the supplementary analysis with SCZ-PRS as the exposure, case vs. control status as the mediator, and globally impaired ERP profile as the outcome reported in the main text, the estimated direct and indirect effects odds ratios (95%CI) before adjusting for the hypothetical unmeasured confounder were 1.76 (0.72, 3.80) and 1.29 (1.12, 1.54), respectively. The effect estimates after adjusting for each hypothetical confounder U are shown in Table S6. Under adjustment of a strong hypothetical confounder with correlations of 0.3 with both mediator and outcome, the estimated direct and indirect effects odds ratios (95%CI) were 1.75 (0.75, 3.99) and 1.24 (1.08, 1.50), respectively, which were very close to the unadjusted effect estimates.

In the supplementary analysis on patients with SCZ-PRS as the exposure, diagnosis (SCZ vs. BPD) as the mediator, and globally impaired ERP profile as the outcome reported in the main text, the estimated direct and indirect effects odds ratios (95%CI) before adjusting for the hypothetical unmeasured confounder were 2.28 (0.94, 6.08) and 1.00 (0.94, 1.10), respectively. The effect estimates after adjusting for each hypothetical confounder U are shown in Table S7. Under adjustment of a strong hypothetical confounder with correlations of 0.3 with both mediator and outcome, the estimated direct and indirect effects odds ratios (95%CI) were 2.47 (0.69,7.07) and 0.99 (0.82, 1.09), respectively, which were very close to the unadjusted effect estimates.

**College-PRS as the exposure:**

In the mediation analysis with college-PRS as the exposure, globally impaired ERP profile as the mediator, and PANSS positive score as the outcome, the estimated direct and indirect effects betas (95%CI) before adjusting for the hypothetical unmeasured confounder were 0.03 (-3.57, 3.69) and 0.90 (0.11, 2.24), respectively. The effect estimates after adjusting for each hypothetical confounder U are shown in Table S8. Under adjustment of a strong hypothetical confounder with correlations of 0.3 with both mediator and outcome, the estimated direct and indirect effects betas (95%CI) were 0.25 (-3.09, 3.83) and 0.49 (0.02, 1.61), respectively. The sensitivity analysis for this mediation analysis indicated that existence of unmeasured confounding would likely lead to overestimation of the indirect effect and underestimation of the direct effect. Nonetheless, the estimated indirect effect remained significant after controlling for a strong hypothetical confounder, and the proportion mediated of 66.5% supported our conclusion that the majority of the effect of college-PRS_PT=0.01_ on PANSS-positive score was indirect.

Sensitivity analyses of unmeasured confounding found similar results after adjusting for hypothetical confounders in the two supplementary mediation analyses with college-PRS as the exposure (Table S9 & S10).

In the supplementary analysis with college-PRS as the exposure, case vs. control status as the mediator, and globally impaired ERP profile as the outcome reported in the main text, the estimated direct and indirect effects odds ratios (95%CI) before adjusting for the hypothetical unmeasured confounder were 4.09 (1.60, 10.59) and 1.19 (1.00, 1.46), respectively. The effect estimates after adjusting for each hypothetical confounder U are shown in Table S9. Under adjustment of a strong hypothetical confounder with correlations of 0.3 with both mediator and outcome, the estimated direct and indirect effects odds ratios (95%CI) were 3.81 (1.40, 10.53) and 1.15 (1.01, 1.41), respectively, which were very close to the unadjusted effect estimates.

In the supplementary analysis on patients with college-PRS as the exposure, diagnosis (SCZ vs. BPD) as the mediator, and globally impaired ERP profile as the outcome reported in the main text, the estimated direct and indirect effects odds ratios (95%CI) before adjusting for the hypothetical unmeasured confounder were 3.94 (1.47, 10.71) and 1.00 (0.93, 1.08), respectively. The effect estimates after adjusting for each hypothetical confounder U are shown in Table S10. Under adjustment of a strong hypothetical confounder with correlations of 0.3 with both mediator and outcome, the estimated direct and indirect effects odds ratios (95%CI) were 4.37 (1.31, 13.55) and 1.02 (0.91, 1.24), respectively, which were very close to the unadjusted effect estimates.

***GWAS of globally impaired ERP***

The GWAS results of globally impaired cluster showed no evidence for genomic inﬂation (lambda-GC of 0.98, Figure S3). Although none of the SNPs reached genome-wide signiﬁcance (P <5E-08), five independent regions including nine SNPs showed suggestive association levels (p < 1E-05) (Figure S4; Table S11). Among the suggestive associated SNPs, rs1424104 and rs4888926 are located in the WWOX gene on chromosome 16, rs4792136 and rs73284773 are located in the SHISA6 gene on chromosome 17, and rs1078008 is located in the VIPR1 gene on chromosome 3. A gain-type copy number variation (CNV) affecting the WWOX gene has been found exclusively in patients with SCZ.^15^ Although the other two genes have not been reported to be associated with psychotic disorders, both are involved in aspects of brain function.

Again, we are aware that the GWAS is underpowered and the results may not be reliable. Therefore, we did not report them in the main text. Replication with larger sample sizes is required.

**SUPPLEMENTARY TABLES**

**Table S1.** **Socio-demographic characteristics of subject groups**

|  | **SCZ Patients** | **BPD Patients** | **Healthy Controls** |
| --- | --- | --- | --- |
|  | N=136 | N=122 | N=125 |
| **Age, yrs** | 44.26(12.36) | 39.81(13.59) | 33.15(12.54) |
| **Female, N (%)** | 90(66.2) | 56(45.9) | 55(44.0) |
| **Education, yrs** | 14.22(2.12) | 14.99(2.30) | 15.61(2.17) |
| **Current Smoker, N (%)** | 51(38.6) | 42(34.7) | 8(6.6) |
| **Age of Onset** | 22.94(7.85) | 22.54(8.86) | -- |
| **CPZ Equivalent Dosage (mg)** | 516.98(577.33) | 214.49(303.28) | -- |
| **PANSS Total** | 62.45(18.79) | 57.96(16.49) | -- |
| **MCAS Total** | 45.15(7.15) | 47.54(5.17) | -- |
| **YMRS Total** | 6.52(8.55) | 10.38(13.42) | -- |
| **MASQ Total** | 141.09(40.88) | 131.75(35.09) | 101.20(21.86) |
| **SHPS** | 1.93(2.34) | 1.58(2.53) | 0.35(0.96) |

Note: Values are means (SD) unless otherwise indicated.

**Table S2. Mean (SD) of ERP measures in each cluster**

| **ERP measures** | **All Subjects** | | |
| --- | --- | --- | --- |
|  | **Globally Impaired**  **N=60** | **Intermediate**  **N=221** | **High Cognitive**  **N=102** |
| **P50 Sensory Gating** | 74.79 (37.76) | 66.95 (42.40) | 37.26 (25.72) |
| **Response to S1** | 2.25 (1.08) | 2.22 (0.88) | 4.45 (1.48) |
| **N1 Amplitude** | -3.64 (2.70) | -3.89 (2.34) | -6.15 (4.00) |
| **P2 Amplitude** | 4.80 (3.00) | 4.68 (2.81) | 9.81 (3.84) |
| **P3 Amplitude** | 5.92 (0.52) | 9.39 (4.26) | 14.12 (5.85) |
| **P3 Latency** | 559.73 (62.43) | 377.19 (36.94) | 364.15 (34.75) |
|  | **Patients Only** | | |
|  | **Globally Impaired**  **N=55** | **Intermediate**  **N=162** | **High cognitive**  **N=41** |
| **P50 Sensory Gating** | 78.55 (37.06) | 73.03 (44.77) | 49.55 (28.42) |
| **Response to S1** | 2.20 (1.10) | 2.22 (0.91) | 4.62 (1.52) |
| **N1 Amplitude** | -3.56 (2.76) | -3.67 (2.24) | -5.83 (4.32) |
| **P2 Amplitude** | 4.95 (3.01) | 4.31 (2.80) | 9.17 (3.50) |
| **P3 Amplitude** | 5.66 (4.00) | 8.70 (4.07) | 12.72 (5.83) |
| **P3 Latency** | 558.56 (62.98) | 380.26 (38.78) | 373.06 (35.39) |

For P50 sensory gating, a lower value indicates better inhibition. For the response to S1, P2 amplitude, and P3 amplitude measures, a higher value indicates larger responses. For the N1 amplitude, a lower value indicates larger responses. For the P3 latency, a lower value indicates faster processing speed.

**Table S3a. Polygenic score association analyses between globally impaired ERP and PRS for each psychiatric or cognitive phenotype in all subjects (n=383)**

| Psychiatric or cognitive phenotype (dataset) | P_T_ | NSNP | R^2^ | Unadjusted P-value | FDR- corrected P-value |
| --- | --- | --- | --- | --- | --- |
| SCZ (PGC) | 0.001 | 2518 | 0.0307 | **0.01** | 0.06 |
|  | 0.01 | 7997 | 0.0108 | 0.12 | 0.23 |
|  | 0.05 | 19823 | 0.0076 | 0.20 | 0.28 |
|  | 0.1 | 29907 | 0.0026 | 0.45 | 0.50 |
|  | 0.5 | 76128 | 5.258E-05 | 0.91 | 0.70 |
| BPD (PGC) | 0.001 | 660 | 0.0005 | 0.73 | 0.63 |
|  | 0.01 | 3827 | 0.0012 | 0.61 | 0.56 |
|  | 0.05 | 13113 | 1.823E-05 | 0.95 | 0.70 |
|  | 0.1 | 22162 | 0.0003 | 0.80 | 0.65 |
|  | 0.5 | 68772 | 0.0013 | 0.60 | 0.56 |
| College Completion (SSGAC) | 0.001 | 730 | 0.0177 | **0.05** | 0.11 |
|  | 0.01 | 4151 | 0.0615 | **2.95E-04** | **0.004** |
|  | 0.05 | 13492 | 0.0291 | **0.01** | 0.06 |
|  | 0.1 | 22246 | 0.0179 | **0.05** | 0.11 |
|  | 0.5 | 64444 | 0.0182 | **0.05** | 0.11 |
| Childhood Intelligence (CHIC) | 0.001 | 314 | 0.0014 | 0.58 | 0.56 |
|  | 0.01 | 2227 | 0.0081 | 0.18 | 0.28 |
|  | 0.05 | 8597 | 0.0240 | **0.02** | 0.08 |
|  | 0.1 | 14828 | 0.0071 | 0.21 | 0.28 |
|  | 0.5 | 47552 | 0.0056 | 0.27 | 0.33 |

P_T_: the P-value threshold used in the training dataset.

NSNP: different number of independent SNPs included for calculating the PRS, which is determined by the selection of PT.

R^2^: Nagelkerke’s pseudo R², the proportion of variance in globally impaired ERP in our study sample explained by the PRS.

Unadjusted P-value: the P-value of the test for association between the PRS and globally impaired ERP, before multiple testing correction

FDR corrected P-value: the P-value after multiple testing (n=20) correction by the FDR q-value method.

All bold values are significant at P <0.05

**Table S3b. Polygenic score association analyses between globally impaired ERP and PRS for each psychiatric or cognitive phenotype in cases with SCZ or BPD (n=258)**

| Psychiatric or cognitive phenotype (dataset) | P_T_ | NSNP | R^2^ | Unadjusted P-value | FDR- corrected P-value |
| --- | --- | --- | --- | --- | --- |
| SCZ (PGC) | 0.001 | 2518 | 0.0176 | 0.09 | 0.17 |
|  | 0.01 | 7997 | 0.0003 | 0.84 | 0.40 |
|  | 0.05 | 19823 | 0.0007 | 0.73 | 0.39 |
|  | 0.1 | 29907 | 0.0002 | 0.87 | 0.40 |
|  | 0.5 | 76128 | 0.0075 | 0.27 | 0.20 |
| BPD (PGC) | 0.001 | 660 | 0.0001 | 0.88 | 0.40 |
|  | 0.01 | 3827 | 0.0077 | 0.26 | 0.20 |
|  | 0.05 | 13113 | 0.0041 | 0.41 | 0.25 |
|  | 0.1 | 22162 | 0.0053 | 0.35 | 0.23 |
|  | 0.5 | 68772 | 0.0010 | 0.69 | 0.39 |
| College Completion (SSGAC) | 0.001 | 730 | 0.0073 | 0.27 | 0.20 |
|  | 0.01 | 4151 | 0.0511 | **0.004** | **0.04** |
|  | 0.05 | 13492 | 0.0234 | 0.05 | 0.16 |
|  | 0.1 | 22246 | 0.0110 | 0.18 | 0.20 |
|  | 0.5 | 64444 | 0.0164 | 0.10 | 0.17 |
| Childhood Intelligence (CHIC) | 0.001 | 314 | 0.0056 | 0.34 | 0.23 |
|  | 0.01 | 2227 | 0.0156 | 0.11 | 0.17 |
|  | 0.05 | 8597 | 0.0370 | **0.01** | 0.06 |
|  | 0.1 | 14828 | 0.0109 | 0.18 | 0.20 |
|  | 0.5 | 47552 | 0.0102 | 0.20 | 0.20 |

P_T_: the P-value threshold used in the training dataset.

NSNP: different number of independent SNPs included for calculating the PRS, which is determined by the selection of P_T_.

R^2^: Nagelkerke’s pseudo R², the proportion of variance in globally impaired ERP in patients of our study sample explained by the PRS.

Unadjusted P-value: the P-value of the test for association between the PRS and globally impaired ERP, before multiple testing correction

FDR corrected P-value: the P-value after multiple testing (n=20) correction by the FDR q-value method.

All bold values are significant at P <0.05

**Table S4a. Results of power calculation for detecting the association between the SCZ-PRS and the globally impaired ERP**

| The genetic correlation between SCZ and globally impaired ERP | Heritability of globally impaired (assumed) | NSNP (assumed) | R^2^ | P-value | Power |
| --- | --- | --- | --- | --- | --- |
| 0.1 | 0.1 | 1000 | 9.8E-04 | 0.26 | 0.08 |
|  |  | 10000 | 8.7E-04 | 0.27 | 0.08 |
|  | 0.3 | 1000 | 0.0030 | 0.19 | 0.14 |
|  |  | 10000 | 0.0026 | 0.20 | 0.13 |
|  | 0.5 | 1000 | 0.0049 | 0.13 | 0.20 |
|  |  | 10000 | 0.0043 | 0.15 | 0.18 |
| 0.3 | 0.1 | 1000 | 0.0089 | 0.07 | 0.33 |
|  |  | 10000 | 0.0078 | 0.08 | 0.29 |
|  | 0.3 | 1000 | 0.027 | 0.005 | 0.75 |
|  |  | 10000 | 0.023 | 0.008 | 0.69 |
|  | 0.5 | 1000 | 0.044 | 3.7E-04 | 0.93 |
|  |  | 10000 | 0.039 | 8.1E-04 | 0.89 |
| 0.5 | 0.1 | 1000 | 0.025 | 0.007 | 0.71 |
|  |  | 10000 | 0.022 | 0.01 | 0.66 |
|  | 0.3 | 1000 | 0.074 | 5.1E-06 | 0.99 |
|  |  | 10000 | 0.065 | 1.9E-05 | 0.99 |
|  | 0.5 | 1000 | 0.12 | 3.0E-09 | 1.00 |
|  |  | 10000 | 0.11 | 2.9E-08 | 1.00 |

NSNP: Different number of independent SNPs included for calculating the PRS, which is determined by the selection of P-threshold.

R^2^: Squared correlation between the PRS and the globally impaired ERP

P-value: Expected p-value of the test for association between the PRS and the globally impaired ERP

Power: The power for detecting the association between the PRS and the globally impaired ERP

**Table S4b. Results of power calculation for detecting the association between the BPD-PRS and the globally impaired ERP**

| The genetic correlation between BPD and globally impaired ERP | Heritability of globally impaired (assumed) | NSNP (assumed) | R^2^ | P-value | Power |
| --- | --- | --- | --- | --- | --- |
| 0.1 | 0.1 | 1000 | 9.3E-04 | 0.27 | 0.08 |
|  |  | 10000 | 5.6E-04 | 0.28 | 0.07 |
|  | 0.3 | 1000 | 0.0027 | 0.19 | 0.13 |
|  |  | 10000 | 0.0017 | 0.23 | 0.10 |
|  | 0.5 | 1000 | 0.0046 | 0.14 | 0.19 |
|  |  | 10000 | 0.0028 | 0.19 | 0.14 |
| 0.3 | 0.1 | 1000 | 0.0084 | 0.08 | 0.31 |
|  |  | 10000 | 0.0051 | 0.13 | 0.21 |
|  | 0.3 | 1000 | 0.025 | 0.006 | 0.72 |
|  |  | 10000 | 0.015 | 0.03 | 0.51 |
|  | 0.5 | 1000 | 0.044 | 2.5E-05 | 0.98 |
|  |  | 10000 | 0.038 | 9.7E-05 | 0.96 |
| 0.5 | 0.1 | 1000 | 0.023 | 0.008 | 0.69 |
|  |  | 10000 | 0.014 | 0.03 | 0.48 |
|  | 0.3 | 1000 | 0.070 | 9.5E-06 | 0.99 |
|  |  | 10000 | 0.042 | 5.0E-04 | 0.91 |
|  | 0.5 | 1000 | 0.12 | 9.0E-09 | 1.00 |
|  |  | 10000 | 0.07 | 8.3E-06 | 0.99 |

NSNP: Different number of independent SNPs included for calculating the PRS, which is determined by the selection of P-threshold.

R^2^: Squared correlation between the PRS and the globally impaired ERP

P-value: Expected p-value of the test for association between the PRS and the globally impaired ERP

Power: The power for detecting the association between the PRS and the globally impaired ERP

**Table S4c. Results of power calculation for detecting the association between the college-PRS and the globally impaired ERP**

| The genetic correlation between college completion and globally impaired ERP | Heritability of globally impaired (assumed) | NSNP (assumed) | R^2^ | P-value | Power |
| --- | --- | --- | --- | --- | --- |
| 0.1 | 0.1 | 1000 | 8.0E-04 | 0.27 | 0.07 |
|  |  | 10000 | 2.9E-04 | 0.30 | 0.06 |
|  | 0.3 | 1000 | 0.0024 | 0.20 | 0.12 |
|  |  | 10000 | 8.7E-04 | 0.27 | 0.08 |
|  | 0.5 | 1000 | 0.0040 | 0.15 | 0.17 |
|  |  | 10000 | 0.0015 | 0.24 | 0.09 |
| 0.3 | 0.1 | 1000 | 0.0072 | 0.09 | 0.28 |
|  |  | 10000 | 0.0026 | 0.20 | 0.13 |
|  | 0.3 | 1000 | 0.022 | 0.01 | 0.66 |
|  |  | 10000 | 0.0079 | 0.08 | 0.30 |
|  | 0.5 | 1000 | 0.036 | 0.001 | 0.87 |
|  |  | 10000 | 0.013 | 0.04 | 0.45 |
| 0.5 | 0.1 | 1000 | 0.020 | 0.01 | 0.63 |
|  |  | 10000 | 0.0073 | 0.09 | 0.28 |
|  | 0.3 | 1000 | 0.060 | 3.7E-05 | 0.98 |
|  |  | 10000 | 0.022 | 0.01 | 0.66 |
|  | 0.5 | 1000 | 0.10 | 9.5E-08 | 1.00 |
|  |  | 10000 | 0.036 | 0.001 | 0.87 |

NSNP: Different number of independent SNPs included for calculating the PRS, which is determined by the selection of P-threshold.

R^2^: Squared correlation between the PRS and the globally impaired ERP

P-value: Expected p-value of the test for association between the PRS and the globally impaired ERP

Power: The power for detecting the association between the PRS and the globally impaired ERP

**Table S4d. Results of power calculation for detecting the association between the childhood intelligence-PRS and the globally impaired ERP**

| The genetic correlation between childhood intelligence and globally impaired ERP | Heritability of globally impaired (assumed) | NSNP (assumed) | R^2^ | P-value | Power |
| --- | --- | --- | --- | --- | --- |
| 0.1 | 0.1 | 1000 | 7.7E-04 | 0.25 | 0.08 |
|  |  | 10000 | 2.5E-04 | 0.29 | 0.06 |
|  | 0.3 | 1000 | 0.0023 | 0.17 | 0.16 |
|  |  | 10000 | 7.6E-04 | 0.26 | 0.08 |
|  | 0.5 | 1000 | 0.0039 | 0.11 | 0.23 |
|  |  | 10000 | 0.0013 | 0.22 | 0.11 |
| 0.3 | 0.1 | 1000 | 0.0070 | 0.06 | 0.37 |
|  |  | 10000 | 0.0023 | 0.17 | 0.15 |
|  | 0.3 | 1000 | 0.021 | 0.002 | 0.81 |
|  |  | 10000 | 0.0068 | 0.06 | 0.37 |
|  | 0.5 | 1000 | 0.035 | 0.0001 | 0.96 |
|  |  | 10000 | 0.011 | 0.02 | 0.56 |
| 0.5 | 0.1 | 1000 | 0.019 | 0.003 | 0.78 |
|  |  | 10000 | 0.0063 | 0.06 | 0.35 |
|  | 0.3 | 1000 | 0.058 | 7.2E--07 | 1.00 |
|  |  | 10000 | 0.019 | 0.004 | 0.78 |
|  | 0.5 | 1000 | 0.097 | 9.4E-11 | 1.00 |
|  |  | 10000 | 0.032 | 0.0002 | 0.94 |

NSNP: Different number of independent SNPs included for calculating the PRS, which is determined by the selection of P-threshold.

R^2^: Squared correlation between the PRS and the globally impaired ERP

P-value: Expected p-value of the test for association between the PRS and the globally impaired ERP

Power: The power for detecting the association between the PRS and the globally impaired ERP

**Table S5. The estimated direct and indirect effect beta (95% CI) of the relationship between the SCZ-PRS, globally impaired ERP profile, and PANSS positive score after adjusting for a hypothetical confounder U**

|  |  | **rUY** | |
| --- | --- | --- | --- |
|  |  | **0.1** | **0.3** |
| **rUM** | **0.1** | βDE= 2.65 (-0.46, 5.46)  βIE= 0.26 (-0.26, 1.35) | βDE= 2.45 (-0.53, 5.39)  βIE= 0.23 (-0.28, 1.25) |
|  | **0.3** | βDE= 2.67 (-0.10, 5.62)  βIE= 0.24 (-0.21, 1.29) | βDE= 2.49 (-0.21, 5.77)  βIE= 0.15 (-0.18, 1.04) |

rUM: the point biserial correlation coefficient between the hypothetical confounder and the mediator

rUY: the Pearson’s correlation coefficient between the hypothetical confounder and the outcome

βDE: direct effect beta

βIE: indirect effect beta

The estimated direct and indirect effects betas (95%CI) before adjusting for the hypothetical unmeasured confounder were 2.68 (-0.37, 5.52) and 0.27 (-0.34, 1.23), respectively.

**Table S6. The estimated direct and indirect effect odds ratio (95% CI) of the relationship between the SCZ-PRS, case-control status, and globally impaired ERP profile after adjusting for a hypothetical confounder U**

|  |  | **rUY** | |
| --- | --- | --- | --- |
|  |  | **0.1** | **0.3** |
| **rUM** | **0.1** | ORDE= 1.76 (0.73, 3.92)  ORIE= 1.29 (1.12, 1.53) | ORDE= 1.74 (0.71, 3.99)  ORIE= 1.27 (1.08, 1.54) |
|  | **0.3** | ORDE= 1.76 (0.74, 3.88)  ORIE= 1.31 (1.12, 1.59) | ORDE= 1.75 (0.75, 3.99)  ORIE= 1.24 (1.08, 1.50) |

rUM: the point biserial correlation coefficient between the hypothetical confounder and the mediator

rUY: the point biserial correlation coefficient between the hypothetical confounder and the outcome

ORDE: direct effect odds ratio

ORIE: indirect effect odds ratio

The estimated direct and indirect effects odds ratios (95%CI) before adjusting for the hypothetical unmeasured confounder were 1.76 (0.72, 3.80) and 1.29 (1.12, 1.54), respectively.

**Table S7. The estimated direct and indirect effect odds ratio (95% CI) of the relationship between the SCZ-PRS, diagnosis (SCZ vs. BPD), and globally impaired ERP profile after adjusting for a hypothetical confounder U**

|  |  | **rUY** | |
| --- | --- | --- | --- |
|  |  | **0.1** | **0.3** |
| **rUM** | **0.1** | ORDE= 2.33 (0.80, 5.46)  ORIE= 1.00 (0.93, 1.09) | ORDE= 2.46 (0.77, 6.73)  ORIE= 1.00 (0.86, 1.05) |
|  | **0.3** | ORDE= 2.33 (0.87, 6.04)  ORIE= 1.00 (0.88, 1.06) | ORDE= 2.47 (0.69,7.07)  ORIE= 0.99 (0.82, 1.09) |

rUM: the point biserial correlation coefficient between the hypothetical confounder and the mediator

rUY: the point biserial correlation coefficient between the hypothetical confounder and the outcome

ORDE: direct effect odds ratio

ORIE: indirect effect odds ratio

The estimated direct and indirect effects odds ratios (95%CI) before adjusting for the hypothetical unmeasured confounder were 2.28 (0.94, 6.08) and 1.00 (0.94, 1.10), respectively.

**Table S8. The estimated direct and indirect effect beta (95% CI) of the relationship between the college-PRS, globally impaired ERP profile, and PANSS positive score after adjusting for a hypothetical confounder U**

|  |  | **rUY** | |
| --- | --- | --- | --- |
|  |  | **0.1** | **0.3** |
| **rUM** | **0.1** | βDE= 0.13 (-3.45, 3.54)  βIE= 0.84 (0.18, 2.23) | βDE= 0.27 (-2.91, 4.04)  βIE= 0.78 (0.14, 2.16) |
|  | **0.3** | βDE= 0.09 (-3.56, 3.77)  βIE= 0.71 (0.11, 2.15) | βDE= 0.25 (-3.09, 3.83)  βIE= 0.49 (0.02, 1.61) |

rUM: the point biserial correlation coefficient between the hypothetical confounder and the mediator

rUY: the Pearson’s correlation coefficient between the hypothetical confounder and the outcome

βDE: direct effect beta

βIE: indirect effect beta

The estimated direct and indirect effects betas (95%CI) before adjusting for the hypothetical unmeasured confounder were 0.03 (-3.57, 3.69) and 0.90 (0.11, 2.24), respectively.

**Table S9. The estimated direct and indirect effect odds ratio (95% CI) of the relationship between the college-PRS, case-control status, and globally impaired ERP profile after adjusting for a hypothetical confounder U**

|  |  | **rUY** | |
| --- | --- | --- | --- |
|  |  | **0.1** | **0.3** |
| **rUM** | **0.1** | ORDE= 4.02 (1.65, 10.35)  ORIE= 1.19 (1.02, 1.48) | ORDE= 3.75 (1.35, 10.87)  ORIE= 1.17 (1.00, 1.45) |
|  | **0.3** | ORDE= 4.06 (1.60, 12.03)  ORIE= 1.21 (1.02, 1.52) | ORDE= 3.81 (1.40, 10.53)  ORIE= 1.15 (1.01, 1.41) |

rUM: the point biserial correlation coefficient between the hypothetical confounder and the mediator

rUY: the point biserial correlation coefficient between the hypothetical confounder and the outcome

ORDE: direct effect odds ratio

ORIE: indirect effect odds ratio

The estimated direct and indirect effects odds ratios (95%CI) before adjusting for the hypothetical unmeasured confounder were 4.09 (1.60, 10.59) and 1.19 (1.00, 1.46), respectively.

**Table S10. The estimated direct and indirect effect odds ratio (95% CI) of the relationship between the college-PRS, diagnosis (SCZ vs. BPD), and globally impaired ERP profile after adjusting for a hypothetical confounder U**

|  |  | **rUY** | |
| --- | --- | --- | --- |
|  |  | **0.1** | **0.3** |
| **rUM** | **0.1** | ORDE= 4.05 (1.48, 10.51)  ORIE= 1.00 (0.93, 1.08) | ORDE= 4.35 (1.38, 13.15)  ORIE= 1.00 (0.92, 1.11) |
|  | **0.3** | ORDE= 4.06 (1.63, 11.53)  ORIE= 1.00 (0.93, 1.13) | ORDE= 4.366 (1.308, 13.554)  ORIE= 1.02 (0.91, 1.24) |

rUM: the point biserial correlation coefficient between the hypothetical confounder and the mediator

rUY: the point biserial correlation coefficient between the hypothetical confounder and the outcome

ORDE: direct effect odds ratio

ORIE: indirect effect odds ratio

The estimated direct and indirect effects odds ratios (95%CI) before adjusting for the hypothetical unmeasured confounder were 3.94 (1.47, 10.71) and 1.00 (0.93, 1.08), respectively.

**Table S11. Suggestive associated SNPs for Globally Impaired ERP**

| CHR | SNP | A1 | A2 | FRQ | OR | SE | P |
| --- | --- | --- | --- | --- | --- | --- | --- |
| 3 | rs1078008 | T | C | 0.7892 | 0.3075 | 0.2534 | 3.27E-06 |
| 6 | rs79617003 | A | G | 0.037 | 9.7991 | 0.4788 | 1.88E-06 |
| 8 | rs62514812 | T | C | 0.9226 | 0.2283 | 0.3321 | 8.70E-06 |
| 8 | chr8_58374581_I | I2 | D | 0.0595 | 5.5597 | 0.3819 | 7.04E-06 |
| 8 | rs10081508 | T | C | 0.0771 | 4.4419 | 0.3358 | 8.96E-06 |
| 16 | rs4888926 | T | C | 0.3311 | 2.7612 | 0.2289 | 9.09E-06 |
| 16 | rs1424104 | T | C | 0.3234 | 2.8247 | 0.23 | 6.35E-06 |
| 17 | rs4792136 | A | C | 0.0752 | 5.098 | 0.355 | 4.48E-06 |
| 17 | rs73284773 | A | G | 0.0718 | 4.8004 | 0.3447 | 5.34E-06 |

**SUPPLEMENTARY FIGURES**


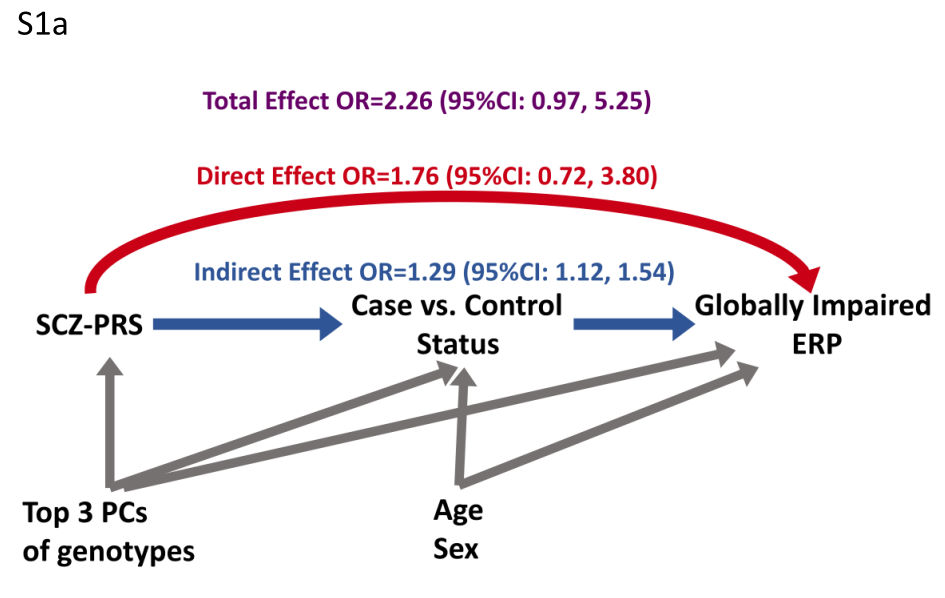
**Figure S1a. Causal Relationship between SCZ-PRS, Case vs. Control Status, and Globally Impaired ERP for all subjects**


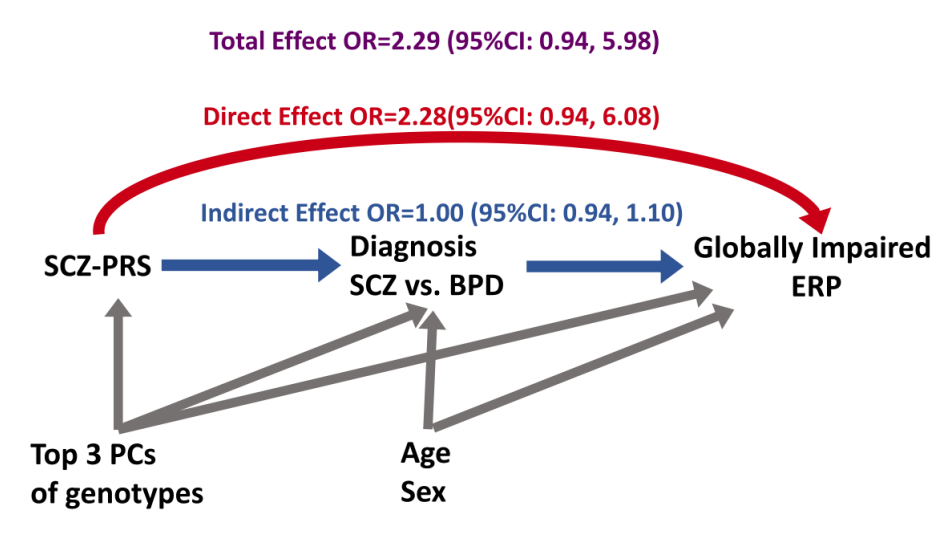
**Figure S1b. Causal Relationship between SCZ-PRS, SCZ vs. BPD Diagnosis, and Globally Impaired ERP for patients**


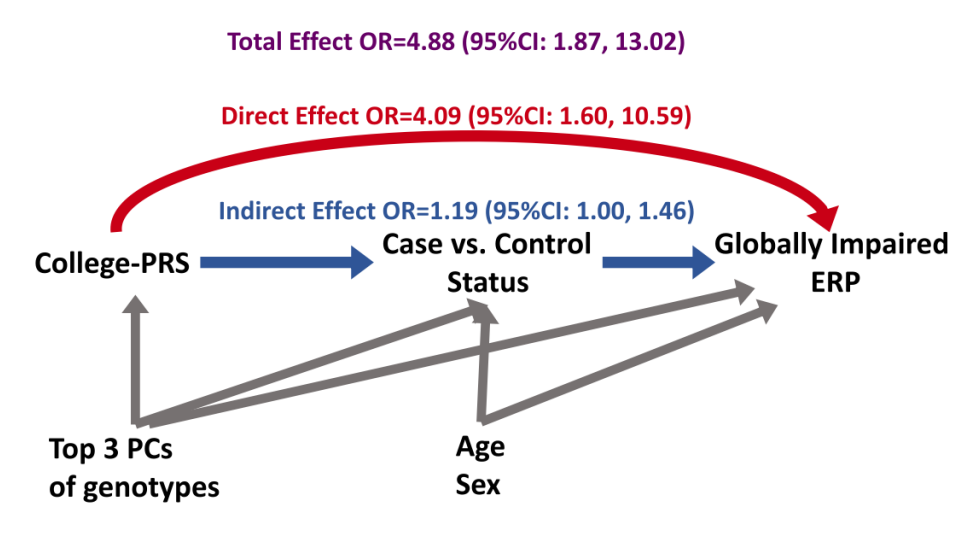
**Figure S2a. Causal Relationship between College-PRS, Case vs. Control Status, and Globally Impaired ERP for all subjects**


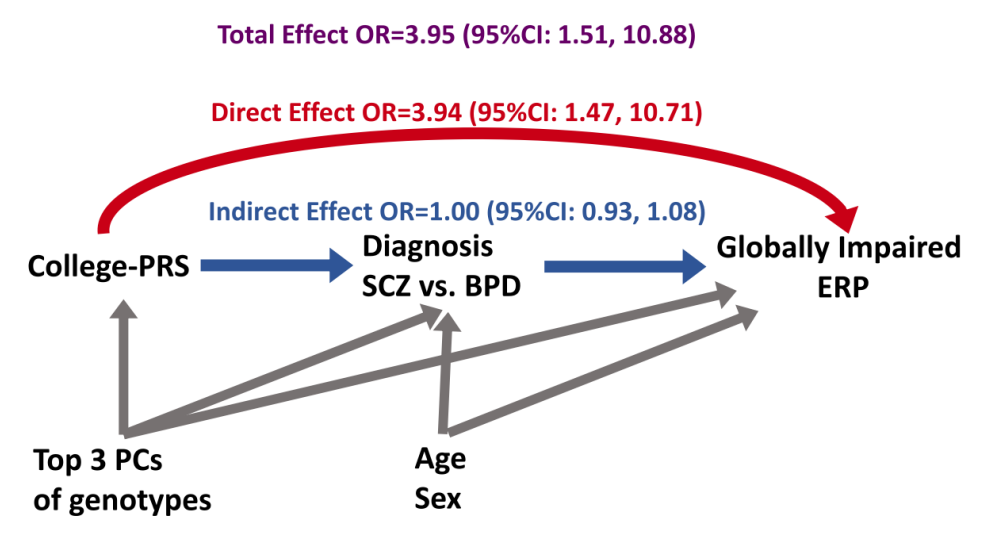
**Figure S2b. Causal Relationship between College-PRS, SCZ vs. BPD Diagnosis, and Globally Impaired ERP for patients**

**Figure S3. Quantile-quantile (Q-Q) plot of observed versus expected P values of the GWAS results for globally impaired ERP profile.**


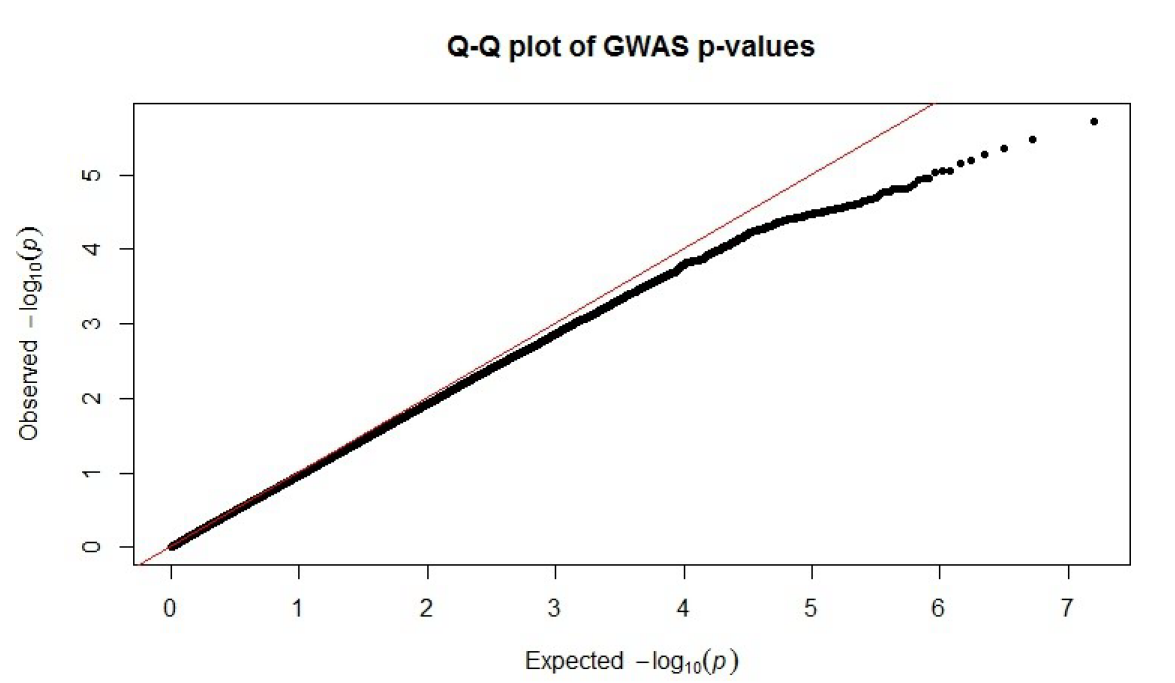


The straight line in the Q-Q plot indicates the distribution of SNPs under the null hypothesis.

**Figure S4. Manhattan plot of the GWAS for globally impaired ERP profile.**


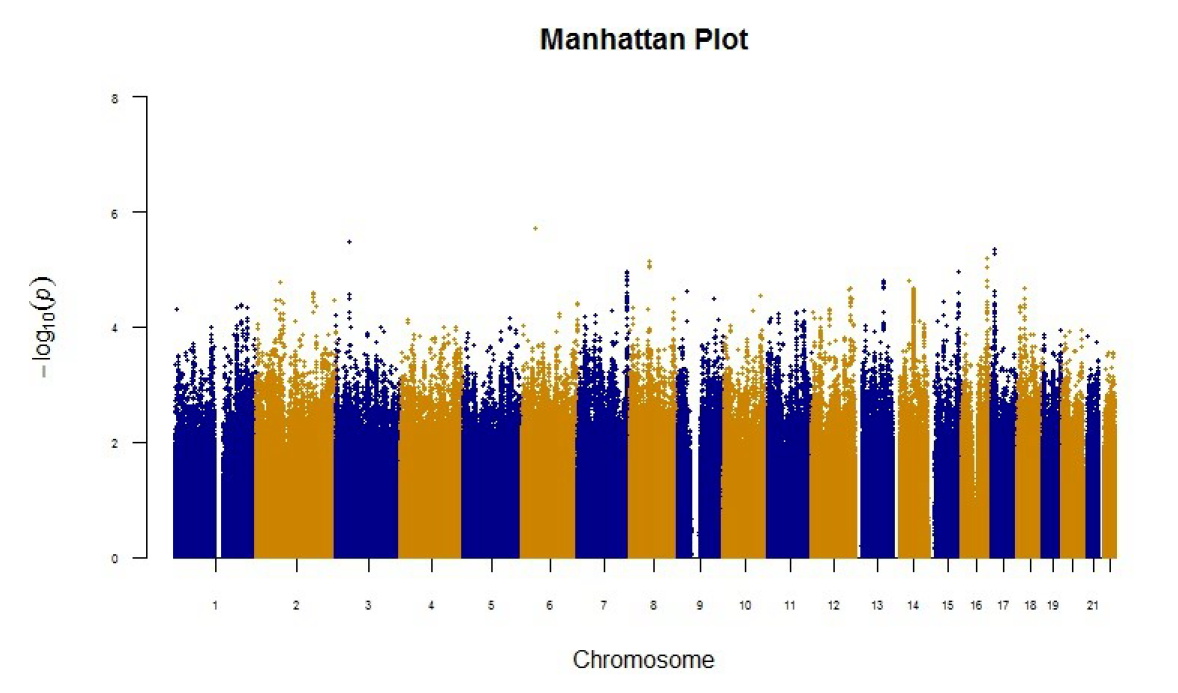


P values (–log10) are plotted against their respective positions on each chromosome.

**Figure S5. Globally impaired ERP may stratify the genetic components of psychotic symptoms.**


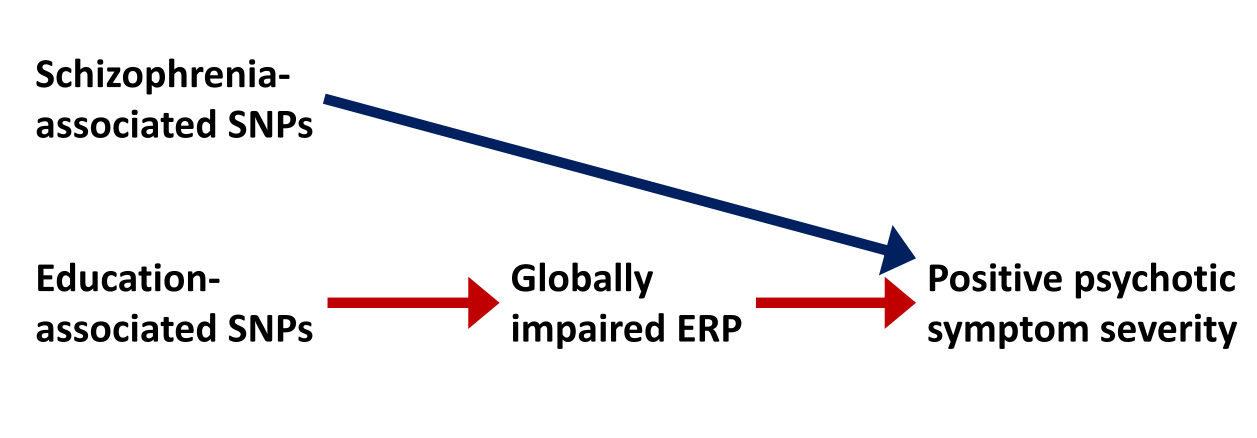


Red arrow: Most of the effect of education-associated SNPs on positive symptom severity is mediated via globally impaired ERP.

Blue arrow: Most of the effect of schizophrenia-associated SNPs on positive symptom severity is through pathways that do not involve globally impaired ERP.

It is possible that SNPs affecting positive symptom severity partially overlap with both SCZ-associated and education-associated SNPs, and globally impaired ERP may capture the component of positive symptoms that is genetically correlated with educational attainment.

**REFERENCES:**

1. Hall MH, Schulze K, Rijsdijk F, Picchioni M, Ettinger U, Bramon E*, et al*. Heritability and Reliability of P300, P50 and Duration Mismatch Negativity. *Behav Genet* 2006; **36**(6)**:** 845-857.

2. Hall MH, Taylor G, Salisbury DF, Levy DL. Sensory gating event-related potentials and oscillations in schizophrenia patients and their unaffected relatives. *Schizophr Bull* 2011; **37**(6)**:** 1187-1199.

3. Gratton G, Coles MG, Donchin E. A new method for off-line removal of ocular artifact. *Electroencephalogr Clin Neurophysiol* 1983; **55**(4)**:** 468-484.

4. Hall MH, Schulze K, Rijsdijk F, Kalidindi S, McDonald C, Bramon E*, et al*. Are auditory P300 and duration MMN heritable and putative endophenotypes of psychotic bipolar disorder? A Maudsley Bipolar Twin and Family Study. *Psychol Med* 2009; **39**(8)**:** 1277-1287.

5. Salisbury DF, Shenton ME, McCarley RW. P300 topography differs in schizophrenia and manic psychosis. *Biol Psychiatry* 1999; **45**(1)**:** 98-106.

6. Salisbury DF, Collins KC, McCarley RW. Reductions in the N1 and P2 auditory event-related potentials in first-hospitalized and chronic schizophrenia. *Schizophr Bull* 2010; **36**(5)**:** 991-1000.

7. Donchin E, Coles MGH. Is the P300 Component a Manifestation of Context Updating. *Behavioral and Brain Sciences* 1988; **11**(3)**:** 357-374.

8. Polich J, Kok A. Cognitive and biological determinants of P300: an integrative review. *Biol Psychol* 1995; **41**(2)**:** 103-146.

9. Dudbridge F. Power and predictive accuracy of polygenic risk scores. *PLoS Genet* 2013; **9**(3)**:** e1003348.

10. Cross-Disorder Group of the Psychiatric Genomics C. Identification of risk loci with shared effects on five major psychiatric disorders: a genome-wide analysis. *Lancet* 2013; **381**(9875)**:** 1371-1379.

11. Okbay A, Beauchamp JP, Fontana MA, Lee JJ, Pers TH, Rietveld CA*, et al*. Genome-wide association study identifies 74 loci associated with educational attainment. *Nature* 2016; **533**(7604)**:** 539-542.

12. Vanderweele TJ. *Explanation in causal inference : methods for mediation and interaction.* . Oxford University Press: New York, 2015.

13. Purcell S, Neale B, Todd-Brown K, Thomas L, Ferreira MA, Bender D*, et al*. PLINK: a tool set for whole-genome association and population-based linkage analyses. *Am J Hum Genet* 2007; **81**(3)**:** 559-575.

14. Price AL, Patterson NJ, Plenge RM, Weinblatt ME, Shadick NA, Reich D. Principal components analysis corrects for stratification in genome-wide association studies. *Nature genetics* 2006; **38**(8)**:** 904-909.

15. Rodriguez-Santiago B, Brunet A, Sobrino B, Serra-Juhe C, Flores R, Armengol L*, et al*. Association of common copy number variants at the glutathione S-transferase genes and rare novel genomic changes with schizophrenia. *Molecular psychiatry* 2010; **15**(10)**:** 1023-1033.
